# Surface Modification of Silica-Supported Supraparticles to Control Their Optical Properties and Mobility in Electric Fields

**DOI:** 10.1021/acs.langmuir.6c00880

**Published:** 2026-06-01

**Authors:** Keisuke Kurioka, Natsuho Tsunetomi, Hikaru Namigata, Keishi Suga, Kanako Watanabe, Daisuke Nagao, Tom A. J. Welling

**Affiliations:** † Department of Chemical Engineering, 13101 Tohoku University, 6-6-07, Aoba, Aramaki-aza, Aoba-ku, Sendai, Miyagi 980-8579, Japan; ‡ School of Engineering, Tohoku University, 6-6-07, Aoba, Aramaki-aza, Aoba-ku, Sendai, Miyagi 980-8579, Japan; § Frontier Research Institute for Interdisciplinary Sciences, Tohoku University, 6-3, Aoba, Aramaki-aza, Aoba-ku, Sendai 980-8579, Japan

## Abstract

Supraparticles, which are spherical assemblies of colloidal particles, have great potential as photonic pigments. Specifically, they have potential as electronic ink particles for switchable display pixels due to their vivid and bright color. However, supraparticles often have low stability, which limits postassembly modification and their mobility in electric fields. In this work, we create robust supraparticles supported by silica via bulk emulsification. The shrinking rate of the droplets was controlled via osmosis to balance the rate of particle assembly, silica formation, and emulsion stability. The silica-supported supraparticles exhibit high order, reflectivity, and suspension stability. Their stability was exploited to modify the surface of the supraparticles with polydopamine (PDA) as the absorbing material to improve saturation. Coating entire supraparticles with PDA yielded similar color saturation compared to supraparticles created using PDA-coated building blocks. Additionally, the supraparticles’ PDA surfaces were further functionalized using polyethylenimine to showcase the possibility of controlling the supraparticles’ surface charge after self-assembly. Finally, the silica-supported supraparticles were moved electrophoretically, which paves the way for these photonic pigments to be used in applications such as reflective electronic ink.

## Graphical abstract

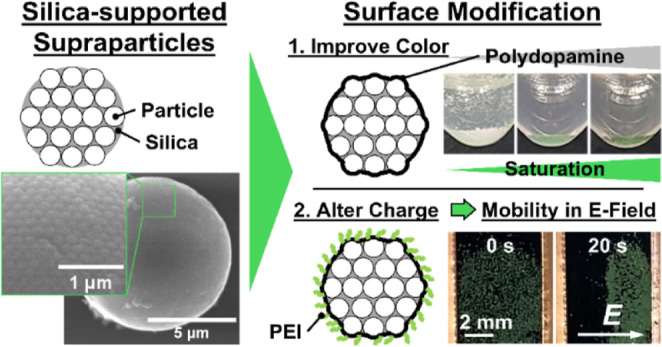

## Highlights

- Photonic silica-supported supraparticles were synthesized via bulk emulsion and osmosis-induced shrinkage.
- Surface modification of silica-supported supraparticles with polydopamine (PDA) achieved color saturation similar to that of supraparticles using PDA-coated building blocks.
- Functionalization of PDA-coated supraparticles enabled control over the surface charge after self-assembly.
- Supraparticles were moved with DC electric fields toward E-ink application.

## Introduction

Colloidal particle assemblies with periodic arrangements exhibit structural colors by diffracting certain wavelengths. These wavelengths can be adjusted by changing the interparticle distance (often using the particle diameter) or the effective refractive index of the structures.^1,2^ Researchers therefore aim to change the properties using external stimuli^3^ such as humidity,^4^ pH,^5^ temperature,^6,7^ mechanical stimuli,^8,9^ magnetic or electric fields,^10,11^ and light.^12,13^ Such tunable colloidal crystals are expected to have applications in anticounterfeiting, sensing, and reflective displays.^11,14–17^ However, these strategies focus on changing the reflection wavelength or intensity,^18^ while barely any research has been performed on moving the entire (small) colloidal crystals in physical space.

Supraparticles, also called supraballs, are spherical assemblies of colloidal particles.^19^ They are typically fabricated using emulsions,^20–23^ hydrophobic^24^ or superamphiphobic surfaces,^25^ or spray-drying.^26,27^ When fabricated using monodisperse particles with diameters between ∼150 and ∼280 nm and shrunk sufficiently slowly, these structures reflect visible light and appear colorful, earning them names such as photonic microbeads or reflective pigments. These photonic supraparticles have been recognized to have potential application in sustainable painting without chemical pigments,^28–30^ passive cooling,^27,31^ anticounterfeiting,^32^ sensing,^33^ and ink-writing and printing.^34,35^

Major challenges for supraparticles include their mechanical stability^36–38^ and suspension stability.^39^ One strategy to increase both mechanical and suspension stability is by growing material in the interstitials of the spherical colloidal crystal.^28,35,40^ Kim et al. produced supraparticles by assembling silica particles in oil-in-water droplets containing poly­(ethylene glycol) phenyl ether acrylate (PEGPEA) and subsequent cross-linking.^35^ This approach yielded elastic photonic microbeads that could be used for direct writing with structural color ink. On the other hand, Clough et al. produced robust photonic pigments by creating spherical inverse opal structures by assembling sacrificial template particles while growing silica between them using sol–gel chemistry.^28^ After removal of the template particles via heat treatment, they obtained water-dispersible photonic pigments. While supraparticles with reinforcing material have been made, they are either soft microparticles (when using polymers)^35^ or lack highly saturated colors.^28^

While surface functionalization is ubiquitous in colloid science to modify surface properties such as charge or hydrophobicity^41,42^ of (nano)­particles, the surface modification of photonic supraparticles is not often conducted as they are unstable in many synthesis environments. Recently, the surface modification of lasing supraparticles, which are excellent laser gaining materials consisting of quantum dots, was conducted to facilitate their dispersion in biological systems.^43^ However, the surface modification of photonic supraparticles to manipulate their optical and surface properties has remained underexplored.

Supraparticles hold great promise as switchable color materials.^44^ Zhang et al. employed poly­(*N*-isopropylacrylamide) (pNIPAM) hydrogel with an inverse opal structure to create supraparticles with a reflectance peak wavelength that depended on temperature.^45^ Kim et al. employed osmotic pressure to tune the volume fraction of particles within a droplet enclosed by a thin shell, leading to changes in the reflection wavelength.^46^ However, structural changes via temperature or osmotic pressure are slow, which is undesirable for switchable color applications such as screens. Others employed magnetic fields to change the orientation of Janus supraparticles to switch between “light” and “dark” states of different colors.^32,47^ Although bulk photonic crystals can respond to electric fields,^48,49^ manipulation of photonic supraparticles using electric fields has not been investigated so far to our knowledge. Switching color between different photonic supraparticles holds great potential for switchable display pixels in reflective E-ink devices.

Both postassembly surface modification of supraparticles and their manipulation in electric fields are severely underexplored. The advantage of postassembly modification lies in the decoupling of the requirements for self-assembly and the requirements for the subsequent application. Self-assembly can be performed with the optimal hydrophilicity and charge of the particles, while surface modification can change the properties afterwards as desired for the application. Therefore, the aim of this study is to demonstrate how surface modification of silica-supported photonic supraparticles after self-assembly could be employed to control their optical and surface properties and their mobility in electric fields. In this work, we use sol–gel chemistry to grow silica between particles during supraparticle formation. This creates photonic silica-supported supraparticles that can be redispersed in water and act as microparticles without being broken apart. As a result, the surfaces of the supraparticles could be modified to improve their optical properties and alter their surface charge. Finally, we show that these charge-controlled supraparticles can be moved electrophoretically, which paves the way for photonic supraparticles as switchable display pixels in reflective e-ink devices.

## Experimental Methods

### Materials

Tetraethyl orthosilicate (TEOS, 95%), ethanol (99.5%), hydrochloric acid (HCl, 0.1 M), potassium peroxodisulfate (KPS, >95.0%), styrene (St, >99.0%), sodium *p*-styrenesulfonic acid (NaSS, >80.0%), sodium hydroxide (NaOH, 1 M), dopamine-hydrochloride (DA-HCl, >98.0%), polyvinylpyrrolidone (PVP, K90 (average molecular weight 360,000)), sodium chloride (NaCl, >99.5%), hexadecane, hexane (>96.0%), and tetraborate pH standard solution (pH 9.18 at 25 °C) were purchased from Fujifilm Wako Chemical Corporation (Osaka, Japan). Span 80 and polyethylenimine (PEI, branched, average molecular weight 25,000) were purchased from Sigma-Aldrich. St was used after removal of the inhibitor using adsorption column equipment. All other chemicals were used as received. Water was deionized in advance (18.2 MΩ cm).

### Synthesis of Polystyrene Particles and Coating Them with PDA

Polystyrene (PSt) particles were synthesized using a soap-free emulsion polymerization method.^50^ The particle size was controlled by adjusting the NaSS concentration (Figures S1 and S2 and Table S1). The polymerization was conducted in a 110 mL glass vial while stirring. First, NaSS was added to deionized water and bubbled with a N_2_ gas for 30 min. St monomer was injected into the vial, and the suspension was stirred at 70 °C for 20 min, after which an aqueous solution of KPS (10.0 mL) was added to initiate the polymerization overnight under a N_2_ atmosphere.

For a typical PDA coating of PSt particles, we used the procedure (Figure S3) from our previous report.^30^ In short, 0.5 vol % PSt particles in water were stirred at room temperature. After 5 min, 0.50 vol % of NaOH aq. (0.1 M) and 1.0 vol % of DA-HCl aq. (1.0 wt %) were added simultaneously. The reaction proceeded for 18 h. The thickness of the PDA coating on the particles can be controlled by adding NaOH and DA-HCl multiple times at 2 h intervals.

### Formation of Silica-Supported Supraparticles

We prepared all aqueous particle dispersions at 20 vol % unless otherwise mentioned. A 40 vol % hydrolyzed TEOS solution in a 1:1 volume ratio of water/ethanol was also prepared by first adding 3.0 mL of ethanol, 2.9 mL of water, and 0.1 mL of 0.1 M HCl aq. to a 20 mL vial. This solution was stirred at room temperature, and after 5 min, 4.0 mL of TEOS was added. The solution was kept stirring for at least 1 h until the liquid became completely transparent.

Two 5 mL tubes were prepared for the synthesis of each supraparticle sample. One tube was used for making water droplets that contained salt (salt droplets), and the other tube was used to make droplets that contained colloidal particles and hydrolyzed TEOS solution (particle/TEOS droplets) (Figure S4). For a typical procedure, in the first tube, 0.3 mL of 0.5 M NaCl aq. was emulsified using 1.5 mL of hexadecane containing 2 wt % Span 80 by shaking with a vortex mixer at 3000 rpm for 30 s. In the second tube, typically 0.075 mL of 40 vol % hydrolyzed TEOS solution and 0.225 mL of an aqueous solution of 20 vol % particles were mixed. Immediately after, 1.5 mL of hexadecane containing 2 wt % Span 80 was added, and the tube was shaken with a vortex mixer at 3000 rpm for 30 s. Salt droplets were added to the tube containing the particle/TEOS droplets. Specifically, using a 1 mL pipet, the salt droplets were added in two aliquots by inserting the pipet in the middle of the liquid and slowly pushing out the salt droplets without bubbling. Then, tubes were left open to let the ethanol evaporate at the oil–air interface after it diffused through the oil. The tubes were left standing for at least 24 h so that the osmosis of water from the particle/TEOS droplets to the salt droplets could take place. Afterward, 0.1 mL of triethylamine was added to the tube to finish gelation of silica for another 30 min.^28^ Subsequently, the samples were washed 3 times with hexane and 3 times with ethanol. The samples were dried overnight in a vacuum oven at room temperature. After the yield was measured, they were redispersed in water to test their stability. (Detailed conditions for each sample are presented in Table S2.)

### Procedure to Measure the Shrinking Rate of Droplets

All particle dispersions were prepared at 10 vol % in deionized water, and all hydrolyzed TEOS solutions were prepared at 20 vol % in a 1:1 volume ratio of water/ethanol beforehand.

Two 2 mL tubes and one shallow cylindrical container were prepared for each sample of the measurement. One tube was used for making water droplets containing salt (salt droplets), and the other tube was used for making water droplets containing colloidal particles and hydrolyzed TEOS solution (particle/TEOS droplets). As for the shallow cylindrical container, the wide end of a 1 mL pipet point was cut and glued onto a cover glass using UV-curing glue to be able to record the shrinking of multiple droplets under an optical microscope. For a typical procedure, in the first tube, 0.05 mL of 2.0 M NaCl aq. was emulsified using 0.25 mL of hexadecane containing 2 wt % Span 80 by shaking with a vortex mixer at 3000 rpm for 30 s. In the second tube, a total of 0.05 mL of aqueous solution was emulsified by mixing the particle dispersion and hydrolyzed TEOS solution with volume ratios of either 1:1, 2:1, or 3:1 and adding 0.25 mL of hexadecane containing 2 wt % Span80, then shaking with a vortex mixer at 3000 rpm for 30 s. Using a 1 mL pipet, salt droplets were added to the tube containing particle/TEOS droplets. Immediately after, 0.05 mL of the oil containing mixed droplets was carefully pipetted into the shallow cylindrical container, and shrinking of droplets was recorded. For each error bar, 3 individual particle/TEOS droplets that did not coalesce with salt droplets were selected, and the diameters were measured by hand using ImageJ, and the diameters were normalized by initial measured diameters.

### Surface Modification of Supraparticles with PDA and PEI

For a typical PDA coating of silica-supported supraparticles, 75 μL of 10 wt % supraparticles in water was pipetted from the stock solution (shortly after being vigorously shaken to obtain a homogeneous concentration of supraparticles) to a 2 mL tube. Then, 1.5 mL of pH 9.18 tetraborate buffer was added to the tube. The tube was set to shake at 1000 rpm at 25 °C using a temperature-controlled shaker (DLab HM100-Pro). After 5 min, the shaking was briefly stopped and an aliquot of freshly prepared 10 wt % DA-HCl in water was added (typically 37.5, 75.0, or 112.5 μL). The shaking was resumed, and the reaction was performed for 20 h, after which the sample was washed 3 times with deionized water to remove the unreacted monomer and PDA from the solution.

To coat the PDA-coated supraparticles with PEI, the supraparticles from the last step were redispersed in 1 mL of pH 9.18 tetraborate buffer containing 10 mg/mL of PEI. The tube was shaken at 1000 rpm at 25 °C for 3 h, after which the sample was washed 3 times with deionized water.

### Characterization

The as-synthesized colloidal particles and supraparticles were observed with a scanning transmission electron microscope (STEM, Hitachi, HD-2700) operated at 200 kV. The supraparticles in water and the shrinking of PSt/TEOS droplets were observed by using an optical microscope (Nikon, Eclipse LV100N POL).

To examine the porosity of the supraparticles, N_2_ adsorption–desorption isotherms were measured with a BELSORP-mini II (Bel Japan Inc.) at 77 K. The pore size distributions were calculated via the Barrett–Joyner–Halenda (BJH) method.

The reflectance spectra were measured at normal incidence via a fiber multichannel spectrometer (SOMA OPTICS, S-2630 Model II) in the wavelength range of 350–1050 nm, equipped with a halogen lamp (SOMA OPTICS, S-2650) at close distance (5 mm). For each sample, 5–10 μL of concentrated supraparticles in water was pipetted onto a glass slide (Matsunami Glass Ind., Ltd., Japan, 76 × 26 mm) that was hydrophilized using a plasma cleaner (Filgen, Ozone Killer). To prevent the sample from spreading, a 3 mm by 3 mm square area was prepared by applying black tape of 200 μm thickness (Vinyl tape Black, Yamato, Inc., Japan) to create an enclosure on the cover glass.

Thermogravimetric analysis (TGA) was conducted via a thermogravimeter-differential thermal analyzer (TG-DTA, Seiko, SSC5200TAstation TG/DTA200). The heating rate was 7 °C min^–1^ from 35 to 800 °C under an air flow of 250 mL min^–1^.

### Analysis of HSV from Photos

To obtain HSV (hue, saturation, value/brightness) values, tubes containing each supraparticle suspension were placed in a test tube stand, and three sides were covered with black cloth to cut off all ambient light except from one direction. Photos of the tubes were taken (Samsung Galaxy S22) from the same angle and ambient light condition for each sample so that saturation and brightness were comparable between images. The saturation and brightness values for each sample were obtained by selecting a square area of the suspension without overexposure using ImageJ, and the mean values were calculated from the histograms and rescaled to a maximum value of 1.0, respectively.

### Electrophoresis Experiments

We fabricated the electric field cell by first placing two pieces of copper tape (3 M Japan, Japan, CU-35C, thickness 0.035 mm) on a cover glass (Matsunami Glass Ind., Ltd., Japan, 24 × 18 mm No.1 (thickness 0.13–0.17 mm)), separated by 6 mm. We then placed a cover glass (Matsunami Glass Ind., Ltd., Japan, 18 × 18 mm No. 1 (thickness 0.13–0.17 mm)) that was hydrophilized using a plasma cleaner (Filgen, Ozone Killer) on top of the electrodes to create a cell with two open ends for liquid exchange. A pipet was used to fill the cell with a solution of 0.1 wt % PVP. After 10 min, deionized water was placed on one end of the cell and filter paper on the other end to replace the PVP that was not adsorbed to the glass by rinsing with water (100 μL). After rinsing with water, the electric field cell was placed in its position for observation (with an optical microscope or a phone camera) and a suspension of supraparticles was introduced into the cell by pipetting.

We used a DC power supply (GW Instek GPS-3030DD, Taiwan) to apply a voltage of 31.2 V, which corresponds to an electric field of 5.2 V/mm. Optical microscope images were recorded at 20× zoom using 1440 × 1024 pixels with 10 fps. Macroscopic videos (Figure 8) were recorded with an iPhone 12 Pro in 4k at 30 fps.

## Results and Discussion

### Fabrication of Silica-Supported Supraparticles

Figure 1 highlights the procedures for synthesizing the supraparticles (SPs) in this work and how their optical and electrical properties are changed by surface modification. As shown in Figure 1a, water droplets containing PSt particles and a hydrolyzed TEOS solution (particle/TEOS droplets) are mixed with aqueous NaCl droplets (salt droplets). The osmotic pressure difference between the two kinds of droplets causes water to migrate from the particle/TEOS droplets to the salt droplets over time. Unless otherwise mentioned, 0.5 M NaCl solution was used for the salt droplets, and the volume ratio between the two kinds of droplets was 1:1. During droplet shrinkage, the particles form a close-packed structure with silica forming between the PSt particles. Since the SPs now act as microparticles, their surface can be modified. Figure 1b shows the two options of adding PDA as an absorbing material to the SPs to enhance color saturation: (1) by coating the PSt building blocks with PDA before SP assembly and (2) by coating the completed silica-supported SPs with PDA after SP assembly. The SP surface can also be further modified to change its surface charge (Figure 1c). In this work, the optical properties and surface charges of the SPs are controlled via these procedures and evaluated.

**Figure 1:**
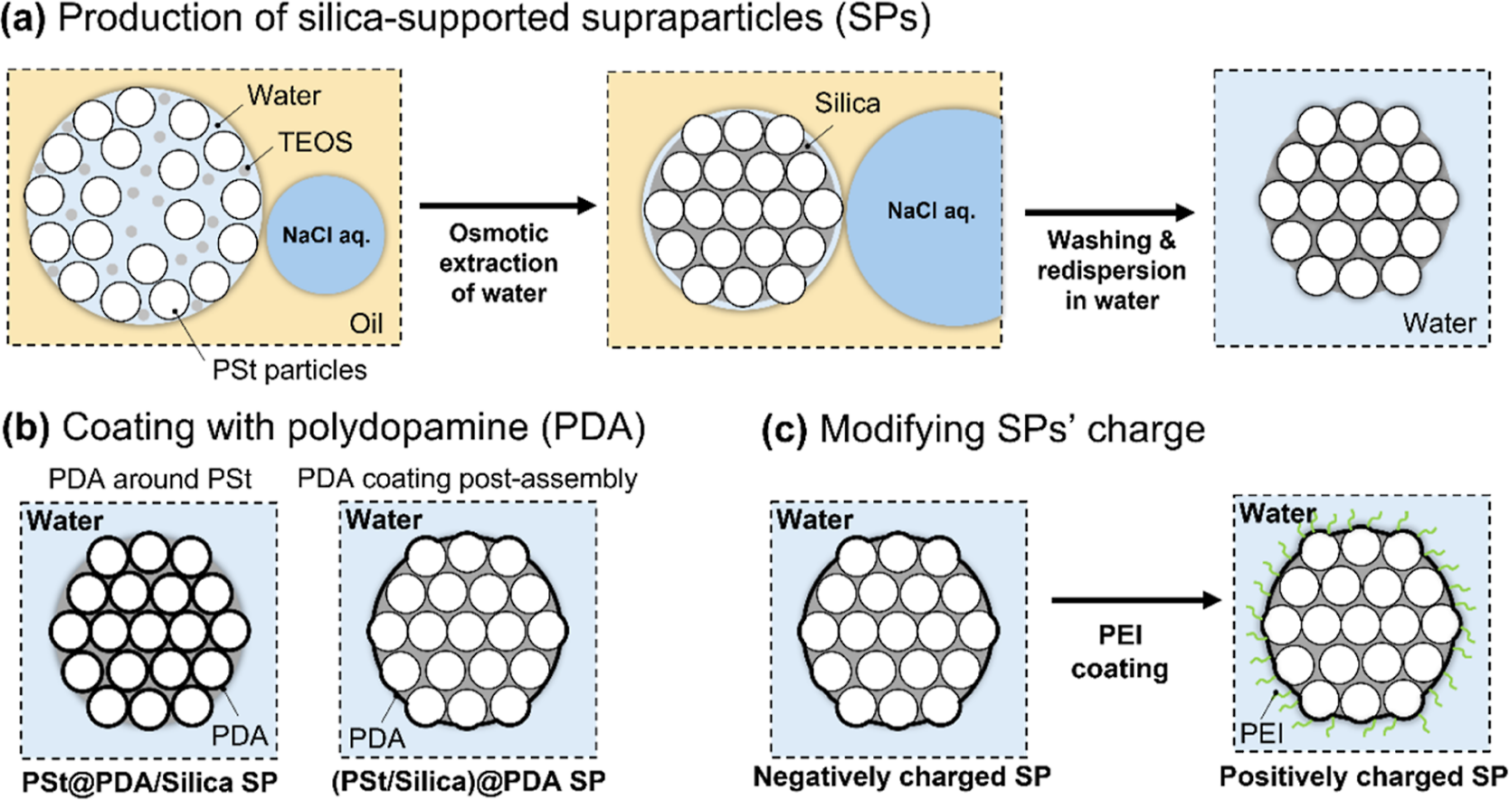
Procedures to (a) form silica-supported supraparticles (SPs), (b) alter their optical properties, and (c) alter their surface charge.

First, the shrinking of particle/TEOS droplets via osmotic extraction is observed in situ under the optical microscope (Figure S5 and Movies S1–S4). Figure 2a indicates the decrease in droplet diameter over time for particle/TEOS droplets of different sizes (at a constant 3:1 volume ratio of 10 vol % particle dispersion to 20 vol % TEOS solution) mixed with 2.0 M NaCl aq. salt droplets. For particle/TEOS droplets, smaller ones shrank slightly faster than larger ones, and most droplets below 80 μm in the initial droplet size had completed shrinking within an hour. Figure 2b shows that the ratio of 10 vol % particle dispersion to 20 vol % TEOS solution hardly influences the shrinking of droplets for initial sizes of 40–60 μm. However, a salt concentration of 0.5 M NaCl in the salt droplets slows down the shrinking dramatically, which is consistent with previous work on SP formation without silica support.^30,51^ For 2.0 M NaCl aq. droplets, the diameter of particle/TEOS droplets had decreased by 30% after 30 min. On the other hand, the diameter of such droplets had decreased by approximately 8% after 30 min in the case of 0.5 M NaCl droplets. Therefore, shrinking with 0.5 M NaCl aq. droplets takes several hours to complete. We conclude that the salt concentration in the droplets predominantly determines the shrinking rate of the droplets, rather than the exact composition of the particle/TEOS droplets. Since the shrinking with 2.0 M NaCl aq. caused many droplets to coalesce, leading to samples with many random aggregates (Figure S6), we employed 0.5 M NaCl aq. salt droplets in the remainder of this work.

**Figure 2:**
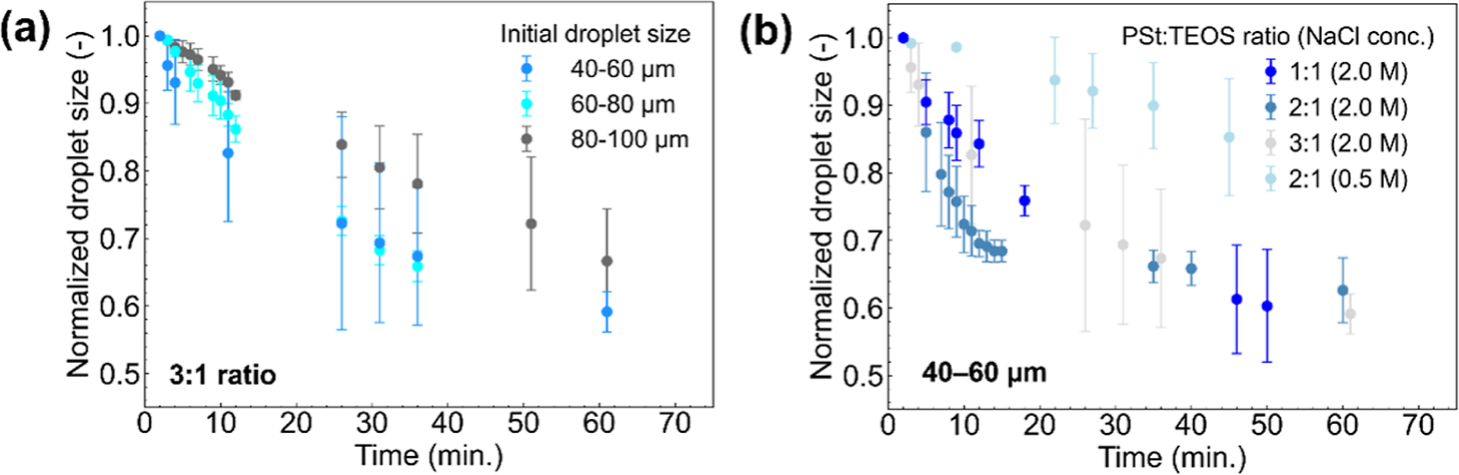
Time-evolution of droplet size during the osmosis-induced shrinking process. (a) Normalized droplet sizes of particle/TEOS droplets for a 3:1 mixing ratio of 10 vol % PSt particle dispersion and 20 vol % hydrolyzed TEOS solution. The droplets were mixed with 2.0 M NaCl aq. droplets in a 1:1 volume ratio. (b) Normalized droplet sizes for different mixing ratios of 10 vol % PSt particle dispersion to 20 vol % hydrolyzed TEOS solution with similar initial droplet sizes (40–60 μm).

In Figure 3, a typical example of silica-supported SPs (PSt/Silica SPs) is shown. Silica is visible between the particles in the micrograph in Figure 3a. An overview SEM image (Figure 3b) indicates that PSt/Silica SPs are mostly spherical with some supraparticles being slightly deformed, which is likely due to early silica gelation. When viewed under the optical microscope, typical features of onion-type and polyhedral assemblies can be observed as shown in Figure 3c. The structure of SPs is remarkable considering that silica was formed between the particles.^28^ The SPs had an average diameter of 22 μm with a high polydispersity due to the bulk emulsification method employed here (Figure 3d). The size of supraparticles, while not significantly altering the reflection peak wavelength, can influence the saturation of a sample because it changes the number of layers of colloidal crystal the light travels through.^16,52^ However, since all samples were made using the same bulk emulsification method, it is unlikely to lead to significant differences between samples. Figure 3e shows the SPs in ethanol showing vivid green colors, which is due to the particle size, *D*_V_ = 206 nm (*C*_V_ = 2.0%), of the building blocks used to fabricate SPs. The silica-supported SPs were found to be stable after shaking in water for 24 h at acidic pH (3.4) and basic pH (10.5), although the silica was found to dissolve at high pH (13.2) as expected.

**Figure 3:**
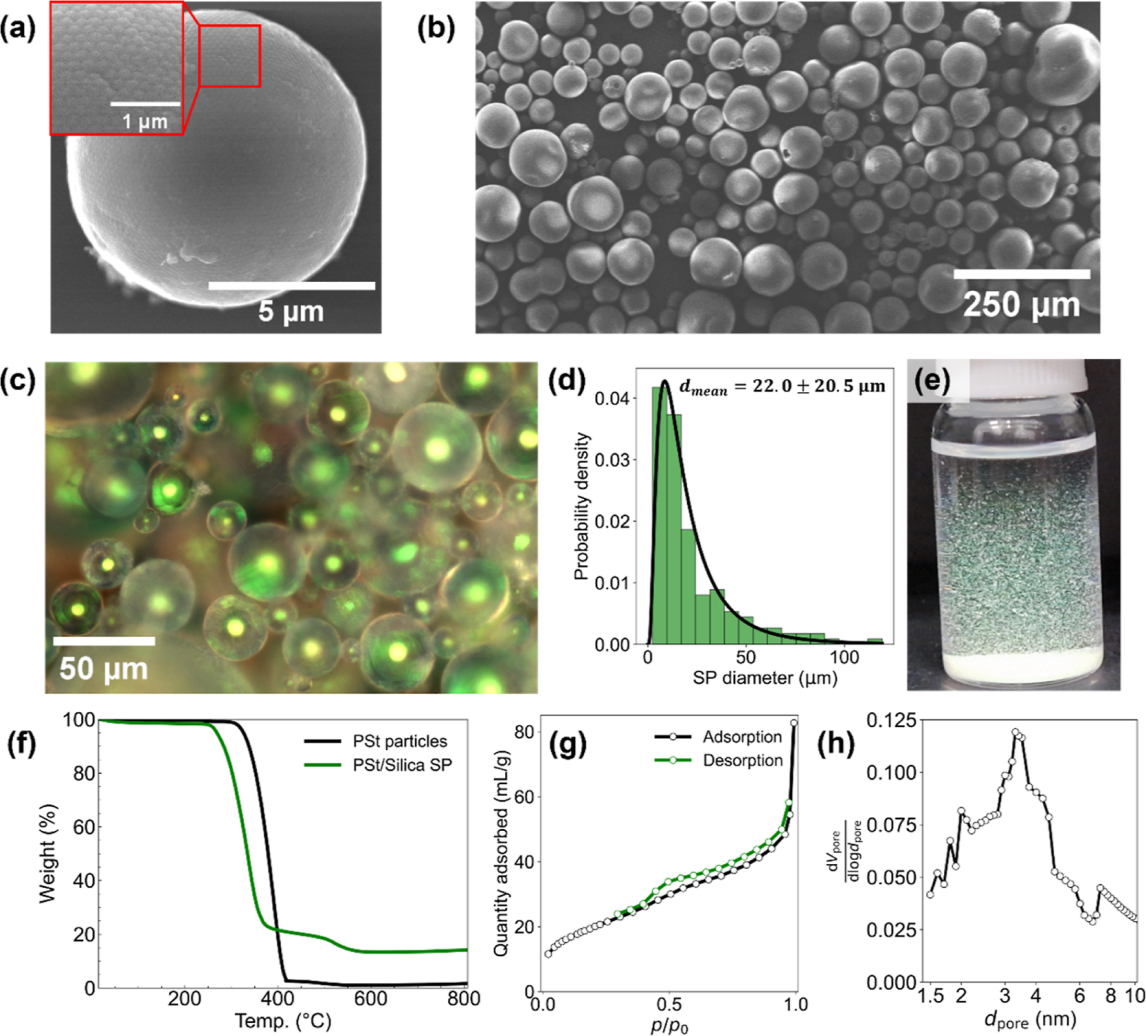
Images and properties of silica-supported supraparticles (PSt/Silica SPs). The SPs were fabricated by mixing an aqueous 20 vol % PSt particle dispersion with a 40 vol % hydrolyzed TEOS solution (30 vol % ethanol, 29 vol % deionized water, 1 vol % 0.1 M HCl aq.) in a 3:1 ratio and emulsified in hexadecane containing 2 wt % Span 80, then were shrunk by mixing the droplets with 0.5 M NaCl aq. droplets. (a) SEM image of a typical PSt/Silica SP. The inset highlights the silica grown at the interstitials of the SP. (b) SEM overview image of many PSt/Silica SPs. (c) Optical microscope image and (d) size distribution of PSt/Silica SPs (*n* = 154). (e) Photograph of PSt/Silica SPs in ethanol. (f) TGA of PSt particles and PSt/Silica SPs. (g) Nitrogen adsorption–desorption measurement hysteresis curves and (h) pore size distribution of PSt/Silica SPs.

Figure 3f displays the TGA of PSt particles and PSt/Silica SPs. It indicates that most of the PSt/Silica SPs’ weight is lost between 300 and 400 °C, which is attributed to PSt pyrolysis. Silica remains at 800 °C, indicating that 14.3 wt % of the sample is silica. Based on the concentrations in the sol–gel reaction and assuming a 100% conversion rate of TEOS to silica, an estimated 14.6 wt % of the sample should be silica, which is in line with the TGA results. Further considering that approximately 30 vol % of an SP is covered by silica (PSt packing fraction of 70 vol %^53^), the silica has a porosity of approximately 78% (derived from PSt and silica densities of 1.05 g/cm^3^ and 1.9 g/cm^3^, respectively), indicating the formation of a highly porous silica gel within SPs.

Figure 3g,h shows the results of N_2_ physisorption of the PSt/silica sample. The adsorption–desorption isotherm hysteresis indicates that there is a mesoporous structure present in the silica backbone with a peak pore diameter of approximately 3.5 nm. The BET surface area was 71 m^2^/g. Considering that 85.7 wt % is PSt and only 14.3 wt % is silica, the surface area of the silica disregarding the PSt particles is approximately 500 m^2^/g, which is typical of a highly porous silica gel. The pore volume was 0.12 cm^3^/g. Once again, considering only the silica weight, this gives a pore volume for the silica gel of 0.84 cm^3^/g. This is in agreement with the sol–gel synthesis of silica microspheres from water droplets containing a small fraction of ethanol as recently reported by Dai and co-workers.^54^

For applications such as printing materials and switchable display pixels, it is desirable to achieve a narrow size distribution while maintaining high packing order and reproducibility of SPs. For further uniform and reproducible packing order of SPs, a more consistent shrinking rate among all droplets could be achieved by having a higher number of NaCl droplets compared to particle/TEOS droplets in the osmosis-induced shrinking process.^51^ As for achieving a narrow size distribution, microfluidics must be used.

Next, we quantified the influence of the ratio between 20 vol % particle dispersion and 40 vol % hydrolyzed TEOS solution on the formation of silica during SP fabrication and their stability. Figure 4a–c shows electron microscopy images of SPs at different volume ratios of 20 vol % particle dispersion and 40 vol % hydrolyzed TEOS solution. At low TEOS content (Figure 4a), silica formation was minimal, yielding SPs that had insufficient silica and were unstable in water; a fraction of these structures fragmented upon redispersion and prolonged agitation in water at 1000 rpm. In contrast, at high TEOS content (Figure 4c), excessive silica resulted in silica-covered SPs. Additionally, only small SPs were present in the final product (Figure 4f), which suggested that large droplets were unstable during the supraparticle formation, likely due to the increased volume of initial ethanol present in the droplets due to the relatively high amount of the hydrolyzed TEOS solution. Instead, droplets that consisted of a 2.5:1 ratio of 20 vol % particle dispersion to 40 vol % hydrolyzed TEOS solution formed SPs with the interstitials filled with silica (Figure 4b). More specifically, the inset shows that the interstitials between the PSt particles are filled with mesoporous silica. In summary, we found that silica-supported SPs synthesized with a 3:1–2:1 ratio between the 20 vol % particle dispersion and the 40 vol % hydrolyzed TEOS solution yielded stable, silica-supported photonic supraparticles.

**Figure 4:**
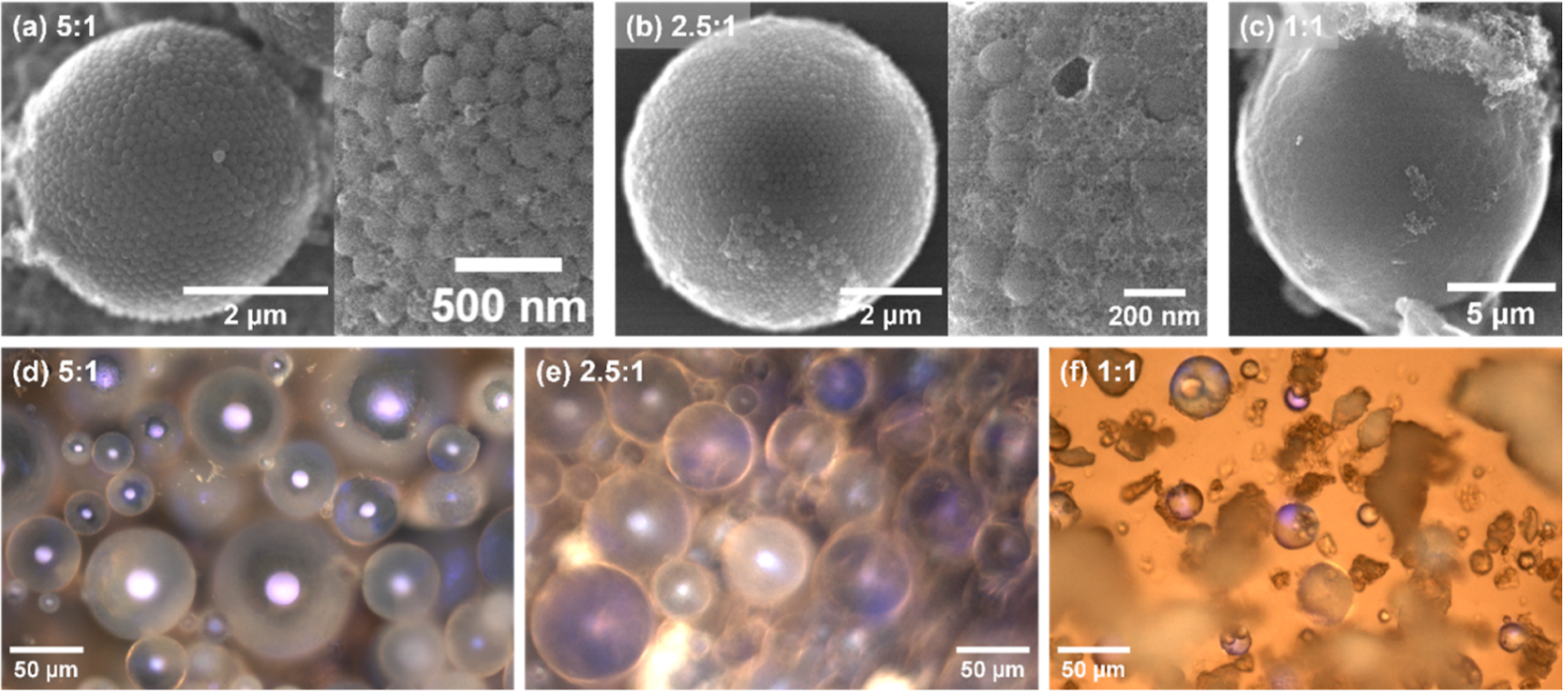
Influence of mixing ratio of 20 vol % particle dispersion to 40 vol % hydrolyzed TEOS solution on the final structure of PSt/Silica SPs. (a–c) SEM images and (d–f) optical microscopy images of PSt/Silica SPs fabricated with mixing ratios of 5:1, 2.5:1, and 1:1, respectively.

### Coating Silica-Supported Supraparticles with PDA

Since silica-supported SPs (PSt/Silica SPs) are stable in water, they are essentially (porous) microparticles with the functionality of a colloidal crystal. This allows us to use surface modification techniques that are often employed for colloidal particles to PSt/Silica SPs instead. As a first step, we coated the PSt/Silica SPs with an absorbing material, PDA, to absorb some of the incoherent scattering that causes the SPs to mostly appear white when viewed at high concentrations. Previous works have shown that adding absorber around the building blocks,^30,55–58^ between the building blocks,^22,29,52^ and within a film containing SPs,^28^ to various degrees, makes samples’ colors more vivid with high saturation. To the best of our knowledge, coating entire SPs with an absorbing material has not yet been shown due to the relative instability of unsupported SPs under coating conditions.

Figure 5a displays photographs of PSt/silica SPs coated with various amounts of PDA ((PSt/silica)@PDA SPs). The sample on the left has no PDA coating, while the sample on the right has been treated with 1.5 mg of DA-HCl per milligram of PSt/silica SPs. The analysis of the photograph in terms of saturation and brightness is presented in Figure 5b. When post-treating the sample with more DA-HCl, the saturation increased while the brightness decreased, which shows successful surface modification without breaking the PSt/silica SPs. Figure 5c shows the reflectance spectra of these SPs. When no PDA coating was conducted, the background increased significantly at shorter wavelengths due to incoherent scattering of PSt particles in the PSt/silica SPs. Absorption increased as the amount of DA-HCl added increased, which can be interpreted by the decrease in background on the shorter wavelengths compared to that on the longer wavelengths, as PDA absorbs more efficiently at shorter wavelengths.^59^ The optimum was found to be 1.0 mg of DA-HCl per mg of PSt/silica SPs, where the background was mostly absorbed while the reflectance peak was not fully absorbed. When increasing the PDA coating further, more of the reflectance peak also gets absorbed, which is indicated by the relatively higher background in the normalized reflectance spectra.

**Figure 5:**
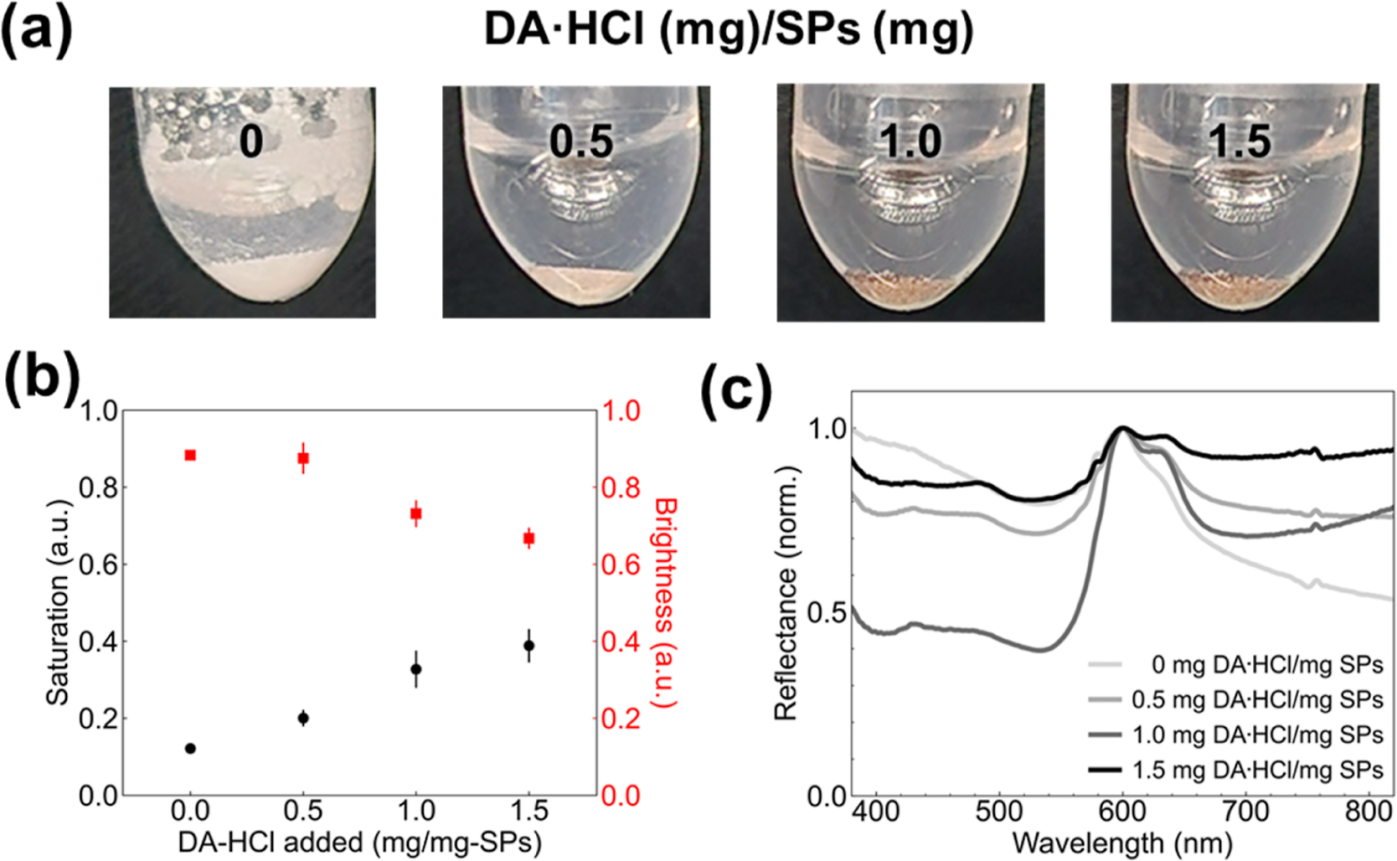
Coating PSt/Silica SPs with PDA ((PSt/Silica)@PDA SPs) by treating different amounts of DA-HCl (mg) per PSt/Silica SPs (mg). (a) Photos of (PSt/Silica)@PDA SP suspension. (b) Saturation and brightness of (PSt/Silica)@PDA SPs derived from photos shown in (a). (c) Reflectance spectra of (PSt/Silica)@PDA SPs.

### Effect of Absorber Location on the Optical Properties of Supraparticles

Figure 6 showcases the differences in optical properties among (PSt/silica)@PDA SPs, where PDA is grown on the silica-supported SPs after assembly, and PSt@PDA/silica SPs, where PDA-coated PSt particles are used to fabricate silica-supported SPs. Porous silica structures within PSt@PDA/silica SPs were confirmed to be similar to those of PSt/silica SPs by N_2_ physisorption analysis and TGA (Figures S7 and S8). Figure 6a–d shows the optical microscopy images of samples with different absorber placements (or none in the case of Figure 6a). Insets show photographs of the SPs in water. Almost all SPs have remarkably high crystalline order, interpreted from the optical reflections and scanning electron microscopy images (Figure S9a,b) with characteristics of a polycrystal,^49^ considering that silica gelated during SP formation. However, the PSt@PDA/silica SPs with the most PDA grown around the PSt building blocks (Figure 6d) had a significantly lower degree of order, as almost all SPs showed optical reflections with characteristics of an onion-like structure, which would reflect stronger in the center and contour of the SP in an isotropic manner.^60,61^ The crystallinity of the obtained SPs in Figure 6a–d was quantitively analyzed by categorizing the SPs by optical reflective characteristics, and the results are shown in Figure 6e. At least 238 SPs for each sample were categorized into low crystalline structures (amorphous) toward more crystalline structures (polycrystal). The PSt/silica SP had the highest number of SPs with large crystalline domains. On the other hand, PSt@PDA-2/silica SP consisted of mostly onion-like structures. This implies that SPs made with PDA-coated PSt building blocks synthesized with a large amount of PDA resulted in fewer crystalline SP structures. The reduced order for SPs made from PDA-coated PSt with a thick PDA coating was confirmed using SAXS and was found to directly correlate with a less pronounced reflectance peak (Figure S13). The values of saturation and brightness of SPs with different absorber placements, obtained from inset images shown in Figure 6a–d, are presented in Figure 6f. (PSt/silica)@PDA SPs with sufficient PDA reach similar values of saturation and brightness to PSt@PDA/silica SPs, despite the absorber being only on the outer surface of the SPs. It is remarkable that similar saturation and brightness can be achieved, as absorber placement was shown to have a significant impact on optical properties of colloidal structures.^62,63^ It is likely that the suboptimal absorber placement on the outside of the SPs is offset by the decreased order for SPs made with PSt@PDA building blocks.^30,55,64^ Moreover, since the silica-supported SPs are porous, it is likely that PDA is present within the SP as well, which would also explain the similar saturation between (PSt/silica)@PDA SPs and PSt@PDA/silica SPs.

**Figure 6:**
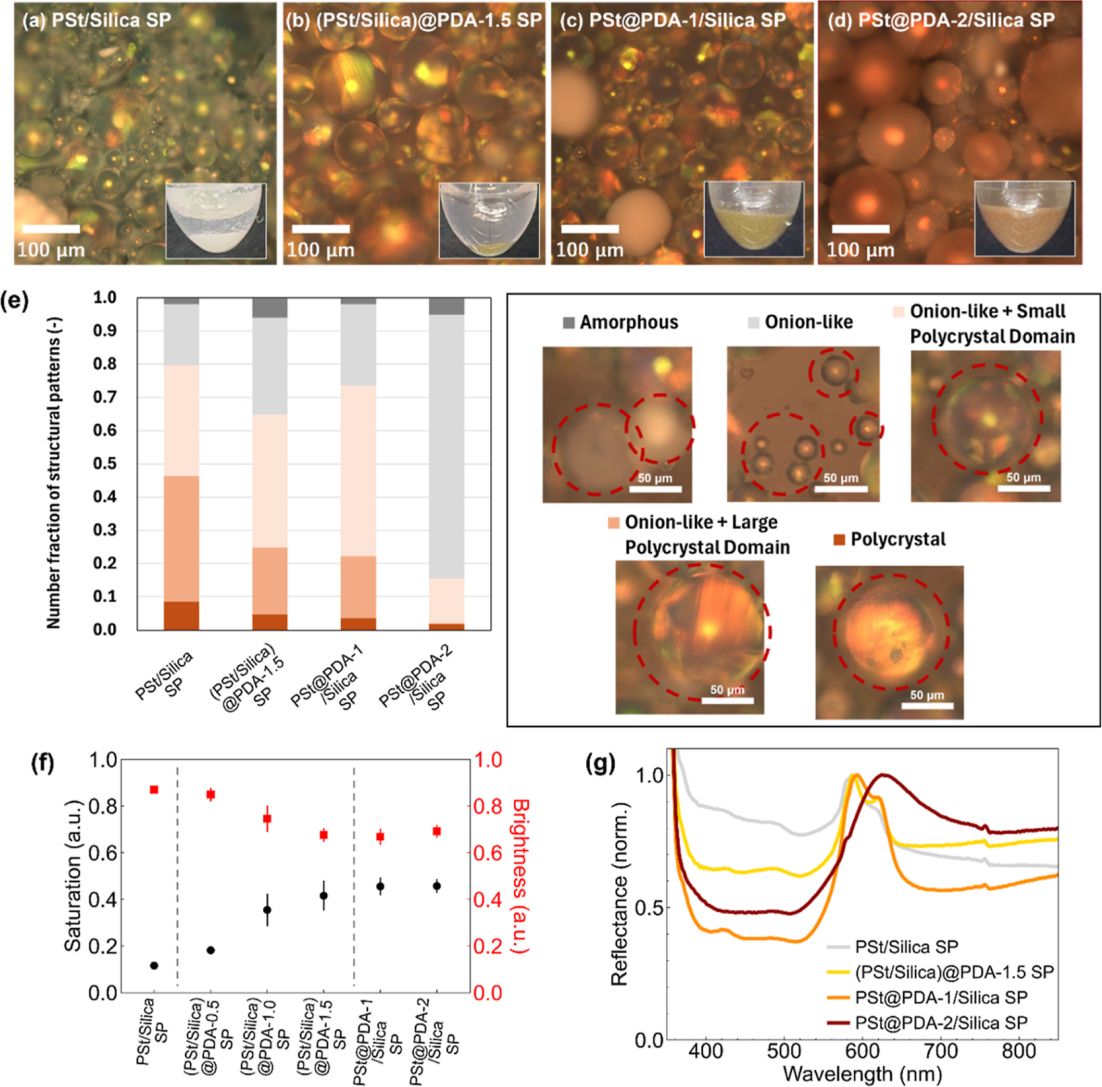
Optical properties of PSt/Silica SPs, PDA-coated SPs ((PSt/Silica)@PDA SPs), and SPs fabricated using PDA-coated PSt particles (PSt@PDA/Silica SPs). (a–d) Optical microscopy images of the SPs with varying PDA placements. Insets show photos of each SP suspension, respectively. (e) Number fraction of supraparticles with a certain type of structure as determined from the optical images in (a–d). (f) Saturation and brightness of each SP derived from photos. (g) Reflectance spectra of each SP.

The reflectance spectra are presented in Figure 6g. A similar trend to Figure 5c is observed where both (PSt/silica)@PDA SPs and PSt@PDA/silica SPs exhibit a lower background at shorter wavelengths compared to PSt/silica SPs (the same trend is observed in Figures S10 and S11). The broader reflectance peak for PSt@PDA-2/silica SPs is likely due to the reduced order of the SPs (Figures 6d and S9c), which could be caused by the thicker dopamine coating (Figures S3, S10c, and S13). Coating the final absorbing layer around the already formed SPs, as is the case for (PSt/silica)@PDA SPs, has the advantage that PDA has no influence on the assembly process.

### PDA Coating for Supraparticles of Different Colors

Figure 7a showcases the different colors obtained with (PSt/silica)@PDA SPs. Figure 7b,c displays the saturation and brightness values derived from the images of (PSt/silica)@PDA SPs fabricated with PSt building blocks of various sizes and the PDA coating using different amounts of DA-HCl after SP assembly. For the (PSt/silica)@PDA SPs with the smallest building blocks (blue SPs), even a small amount of DA-HCl leads to rapidly decreasing brightness, while saturation hardly increases (also refer to Figure S10a,b). On the other hand, green, yellow, and orange SPs have increasing saturation while maintaining relatively high brightness at higher concentrations of DA-HCl. These results can be explained by recognizing that PDA absorbs most efficiently at shorter wavelengths.^59^ (PSt/silica)@PDA SPs reached saturation and brightness values similar to those of PSt@PDA/silica SPs for all colors (Figure S12).

**Figure 7:**
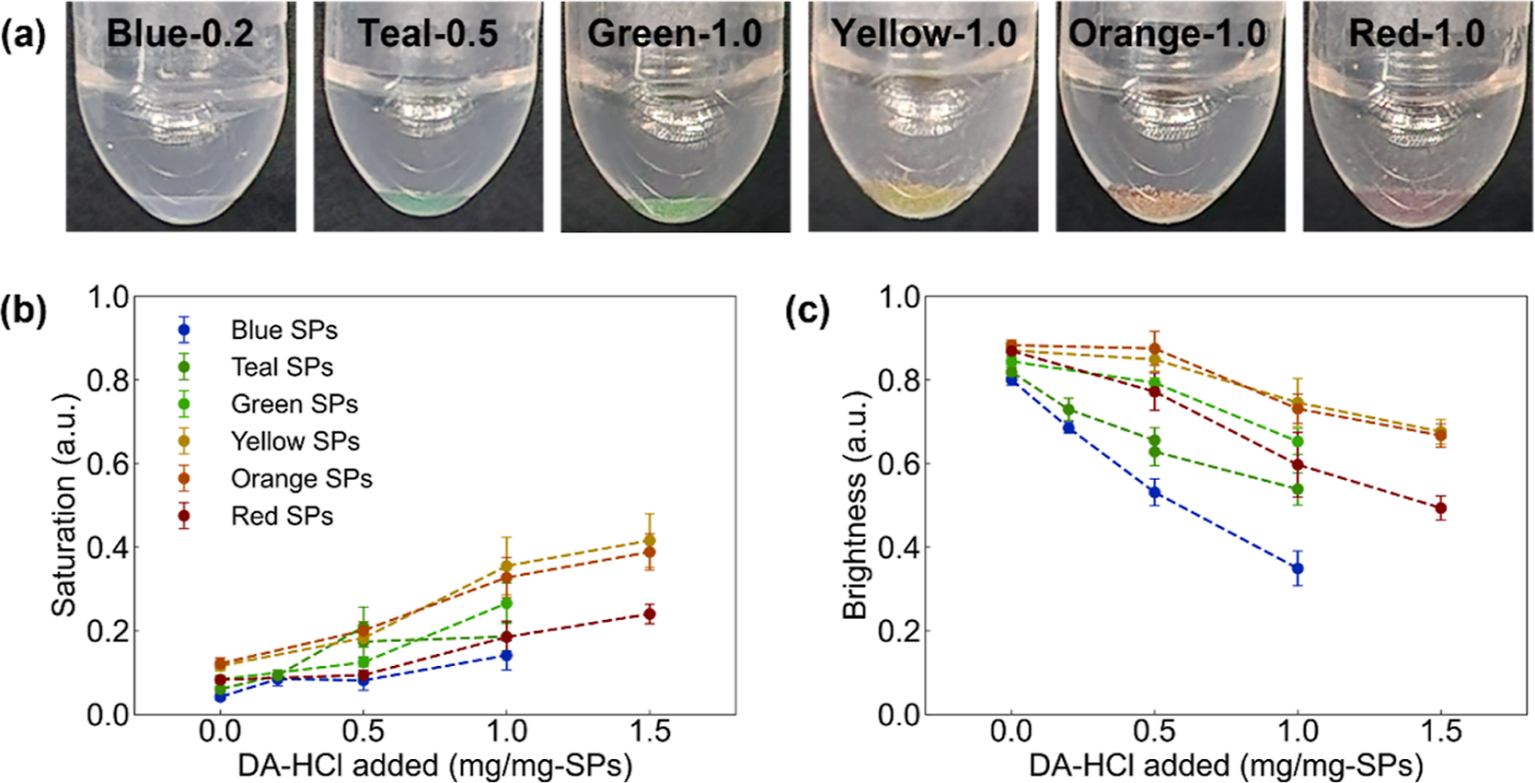
Optical properties of various (PSt/Silica)@PDA SPs fabricated using PSt particles of different sizes and PDA-coating after SP assembly. (a) Photos of (PSt/Silica)@PDA SP suspension. The number designated after each color label corresponds to the amount of DA-HCl (mg) per PSt/Silica SPs (mg) treated for each PDA coating. (b) Saturation and (c) brightness of (PSt/Silica)@PDA SPs derived from photos. Different colors were obtained by different sizes of PSt building blocks used to fabricate SPs: blue (*D*_V_ = 161 nm), teal (*D*_V_ = 201 nm), green (*D*_V_ = 206 nm), yellow (*D*_V_ = 231 nm), orange (*D*_V_ = 238 nm), and red (*D*_V_ = 273 nm).

### Modifying Supraparticles’ Charge and Their Mobility in an Electric Field

In the previous section, we showcased how post-treatment can alter the optical properties of silica-supported SPs. Surface modification is often used to alter the surface charge of colloidal particles. Since silica-supported SPs effectively act as microparticles, the surface of (PSt/Silica)@PDA SPs was further modified with PEI via the Michael addition and Schiff base reactions between nucleophilic amines of PEI and quinone groups of PDA,^65–67^ which turns the negatively charged surfaces of SPs ((PSt/Silica)@PDA SPs) into positively charged surfaces ((PSt/Silica)@PDA@PEI SPs), in water.^66^ However, the zeta potential of SPs is not easily measurable due to their large size, which makes conventional electrophoretic light scattering measurements problematic. As such, the electrophoretic mobility of various SPs was measured via optical microelectrophoresis (particle-tracking electrophoresis under an applied DC field).

Figure 8a shows optical microscope snapshots of a movie (Movie S5) where (PSt/silica)@PDA@PEI SPs in deionized water are controlled by a 5.2 V/mm DC electric field. The (PSt/silica)@PDA@PEI SPs migrate to the negative electrode without breaking apart as evidenced by their enduring structural color. This underlines their robustness and successful coating with cationic PEI. In contrast, PSt@PDA/silica SPs and (PSt/silica)@PDA SPs moved toward the positive electrode, revealing negatively charged surfaces (Figure 8b and Movies S6 and S7). The electrophoretic mobility measured for the different types of supraparticles (Figure 8b) was not converted to a zeta potential of the supraparticles due to the uncertainties in the measurement. Specifically, hydrodynamic drag near the walls of the electric-field cell could significantly slow down the mobility of the supraparticles, which makes the conversion to a zeta potential nontrivial.

**Figure 8:**
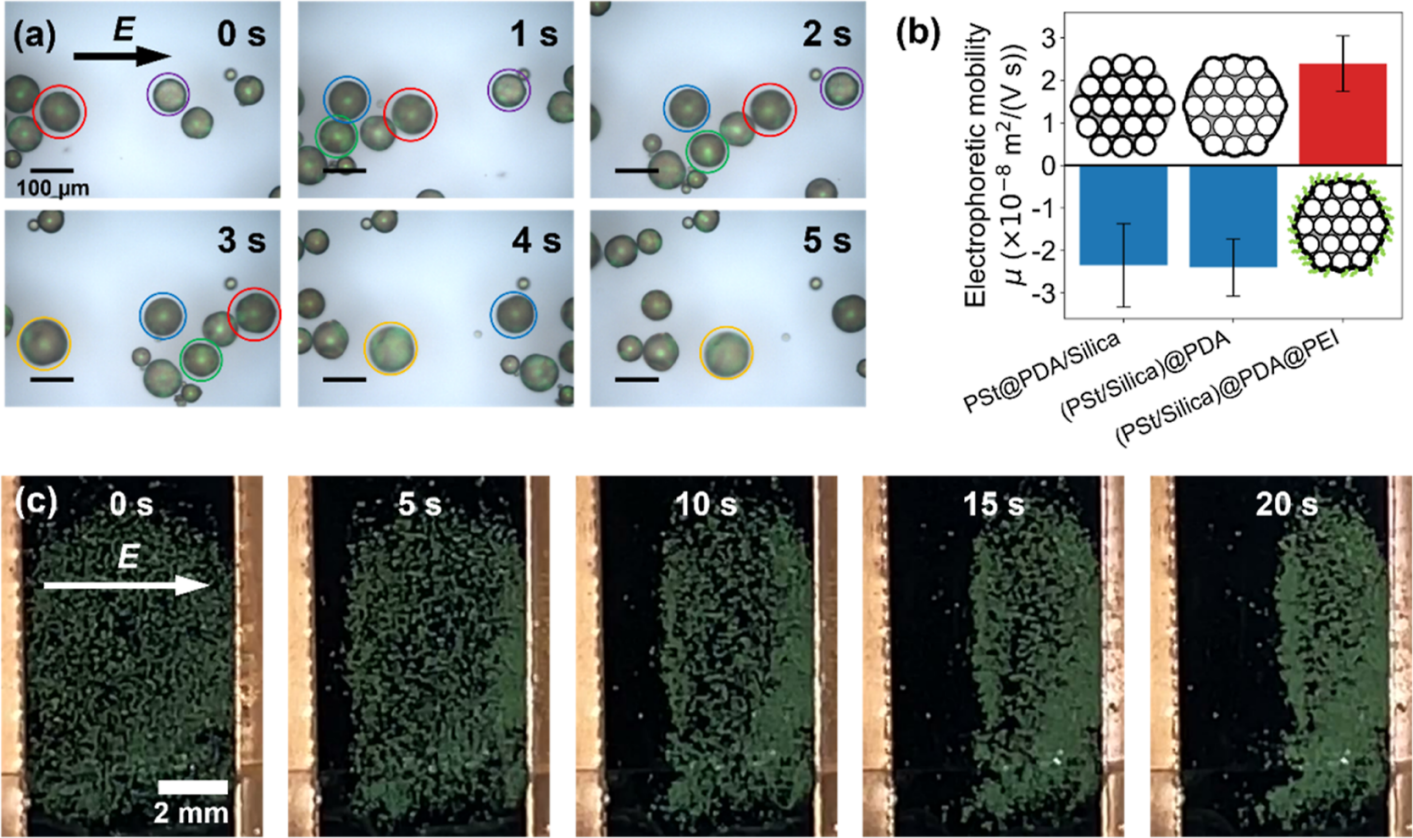
Electrophoretic mobility of SPs in a DC electric field filled with deionized water. (a) Frames of a microscopic video of (PSt/Silica)@PDA@PEI SPs moving in a 5.2 V/mm DC electric field (Movie S5). Some SPs are encircled for easier identification. (b) Electrophoretic mobility of different SPs measured via particle tracking (Movies S5–S7). (c) Frames of a macroscopic video shot in ambient light setting, showing the positively charged (PSt/Silica)@PDA@PEI SPs moving toward the negative electrode (Movie S8).

Finally, we performed electric field experiments with a higher concentration of (PSt/silica)@PDA@PEI SPs to investigate whether we can observe the migration of photonic SPs on a macroscopic scale. Figure 8c shows snapshots of a video of (PSt/silica)@PDA@PEI SPs moving in a 5.2 V/mm DC electric field under ambient light. The macroscopic migration of the photonic SPs can be clearly observed, which was reversible (Movie S8). These results show that silica-supported SPs can have their charge altered after self-assembly and can be moved in electric fields while maintaining their structural color, which opens the path to using photonic SPs in switchable display pixels for reflective E-ink screens.

## Conclusions

This work addressed the dispersion stability of photonic supraparticles and highlighted the potential of surface modification to manipulate the supraparticles’ optical and electrical properties. Silica-supported supraparticles containing arrangements of PSt particles were fabricated via bulk emulsification of an aqueous phase containing colloidal particle dispersion and hydrolyzed TEOS solution. The droplets were shrunk osmotically over the course of few hours. A ratio of 20 vol % particle dispersion to 40 vol % TEOS solution between 3:1 and 2:1 was found to be ideal to fabricate supraparticles supported by a silica.

The silica-supported supraparticles were post-treated with PDA to alter their optical properties. The color saturation of silica-supported supraparticles, especially those with reflectance peaks at higher wavelengths, was improved by coating the entire supraparticle with PDA while maintaining a relatively high brightness. Additionally, the PDA-coated supraparticles were further modified with PEI to change their surface charges. Moreover, when applying an electric field, the photonic supraparticles moved electrophoretically, which was observable by eye as photonic microparticles moved toward one of the electrodes.

These results show the power of postprocessing silica-supported supraparticles using surface modification. It allows us to decouple the self-assembly process from the properties needed for the intended application. Since the supporting silica is highly porous, this does not exclude applications such as adsorption^68^ or catalysis.^69^ We especially highlight the possibility of using photonic supraparticles as switchable display pixels for electronic ink applications.^70^ To this end, silica-supported supraparticles should be fabricated via microfluidics to control their size and their self-assembly should be improved to predominantly produce icosahedral structures while silica gel is grown between the particles. By subsequently modifying the high-saturation supraparticles’ surface with molecules that impart charge on them in low dielectric media, they could be used in reflective screens.

## Supplementary Material



















## Notes

Timelapses of shrinking droplets, optical microscopy videos of electrophoretically moving silica-supported supraparticles, and a macroscopic video of the migration of structurally colored silica-supported supraparticles in an electric field (MP4)
(MP4)
(MP4)
(MP4)
(MP4)
(MP4)
(MP4)
(MP4)
Schematics depicting the synthesis procedure for PSt particles, PSt@PDA particles, and silica-supported SPs; table of exact synthesis conditions of each supraparticle ordered by main-text figure number; frames of optical microscope video of in situ osmotic shrinking of droplets; images of SPs fabricated using 2.0 M NaCl aq. salt droplets; physisorption and TGA of (PSt@PDA)/Silica SPs; saturation and brightness measurements of (PSt@PDA)/Silica SPs; reflectance spectra of silica-supported SPs comparing the wet and dry states; additional optical properties of silica-supported properties with smaller PSt building blocks coated with PDA (reflectance wavelengths around 440 nm); SAXS measurements of (PSt/Silica)@PDA SPs and PSt@PDA/Silica SPs for comparison to the reflectance spectra; zeta potential of PSt and PSt@PDA particles; images of (PSt/Silica)@PDA SPs and PSt@PDA/Silica SPs after shaking in various pH conditions (PDF)
