# Supplementary material for: Surface Modification of Silica-Supported Supraparticles to Control Their Optical Properties and Mobility in Electric Fields
Source: Langmuir. 2026 Jun 1;42(23):16327–38. doi: 10.1021/acs.langmuir.6c00880 (PMC13276887; doi:10.1021/acs.langmuir.6c00880)
Supplement: Supplementary file 9 [file la6c00880_si_009.pdf]

Surface modification of silica-supported supraparticles to control their optical properties and mobility in electric fields

Keisuke Kurioka,[1] *Natsuho Tsunetomi*[2,3]*, Hikaru Namigata,*[1]

Keishi Suga,[1] *Kanako Watanabe,*[1] *Daisuke Nagao*[1]*, and Tom A.J. Welling,*[1,3,*]

1: Department of Chemical Engineering, Tohoku University

6-6-07, Aoba, Aramaki-aza, Aoba-ku, Sendai, Miyagi, 980-8579, Japan

2: School of Engineering, Tohoku University

6-6-07, Aoba, Aramaki-aza, Aoba-ku, Sendai, Miyagi, 980-8579, Japan

3: Frontier Research Institute for Interdisciplinary Sciences, Tohoku University

6-3, Aoba, Aramaki-aza, Aoba-ku, Sendai 980-8579, Japan

* Corresponding author

Tom A. J. Welling (E-mail: t.a.j.welling@tohoku.ac.jp, TEL: +81 22-795-7240)

Number of pages: 19

Number of figures: 16

Number of schemes: 0

Number of tables: 2

Tables of contents

Synthesis of polystyrene (PSt) particles 3

Figure S1. Synthesis procedure for PSt particles. 3

Table S1. Particle diameter and coefficient of variation of each particle. 4

Figure S2. TEM images of PSt particles from Table S1. 4

Synthesis of PSt@PDA particles 4

Figure S3. Procedure for coating PSt particles to make PSt@PDA particles. 4

Procedure to produce silica-supported supraparticles 5

Figure S4. Procedure for silica-supported supraparticle production. 5

Table S2. Synthesis conditions for each supraparticle sample. 5

Shrinking droplets with 2.0 M NaCl aq. droplets 8

Figure S5. Optical microscope snapshots during osmotic shrinking. 8

Figure S6. TEM and optical microscope images of supraparticles (2.0 M NaCl). 9

Physisorption & thermogravimetric analysis of PSt@PDA/Silica SPs 10

Figure S7. Nitrogen physisorption results and pore size distribution. 10

Figure S8. TG results for PSt, PSt/Silica, PSt@PDA, and PSt@PDA/Silica SPs. 10

Structural and Optical Characterization 11

Figure S9. TEM images of (PSt/Silica) and PSt@PDA/Silica SPs. 11

Figure S10. Optical properties of SPs with varying PDA coatings. 12

Figure S11. Reflectance spectra of SPs in the wet and dry state. 12

Figure S12. Saturation and brightness for PSt@PDA/Silica SPs. 13

SAXS Analysis and Zeta Potential 13

Figure S13. SAXS and reflectance measurements of various SPs. 14

Zeta potential of PSt & PSt@PDA building blocks 15

Figure S14. Zeta potential of PSt and PSt@PDA building blocks. 15

Stability Evaluation in Different pH 16

Figure S15. Optical images of SPs after shaking at various pH conditions. 16

Figure S16. SEM images of PSt@PDA/Silica SPs after pH stability tests. 17

Supporting Movie Captions 18

References 19

Synthesis of polystyrene (PSt) particles

Monomer:	Styrene	500 mM
Co-monomer:	NaSS	X mM
Initiator:	KPS	10 mM
Solvent:	Deionized water	

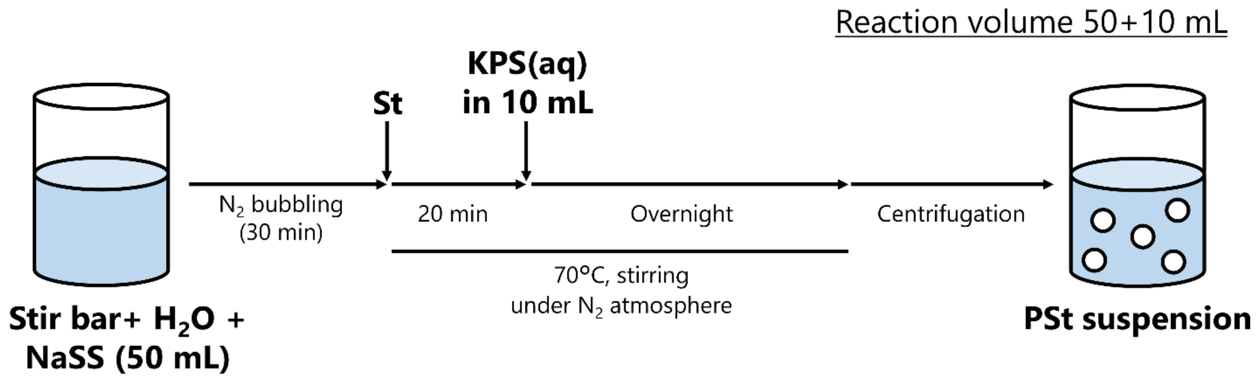

Figure S1. Synthesis procedure for PSt particles.

Table S1. Particle diameter and coefficient of variation of each particle. *Synthesis performed with lower total volume.

Sample name	D_V [nm]	C_V [%]	NaSS [mM]
PSt_1	161	6.8	1.2
PSt_2	201	2.4	0.9
PSt_3	206	2.0	0.9
PSt_4	231	1.5	0.75
PSt_5	238	1.9	0.55*
PSt_6	273	1.8	0.6

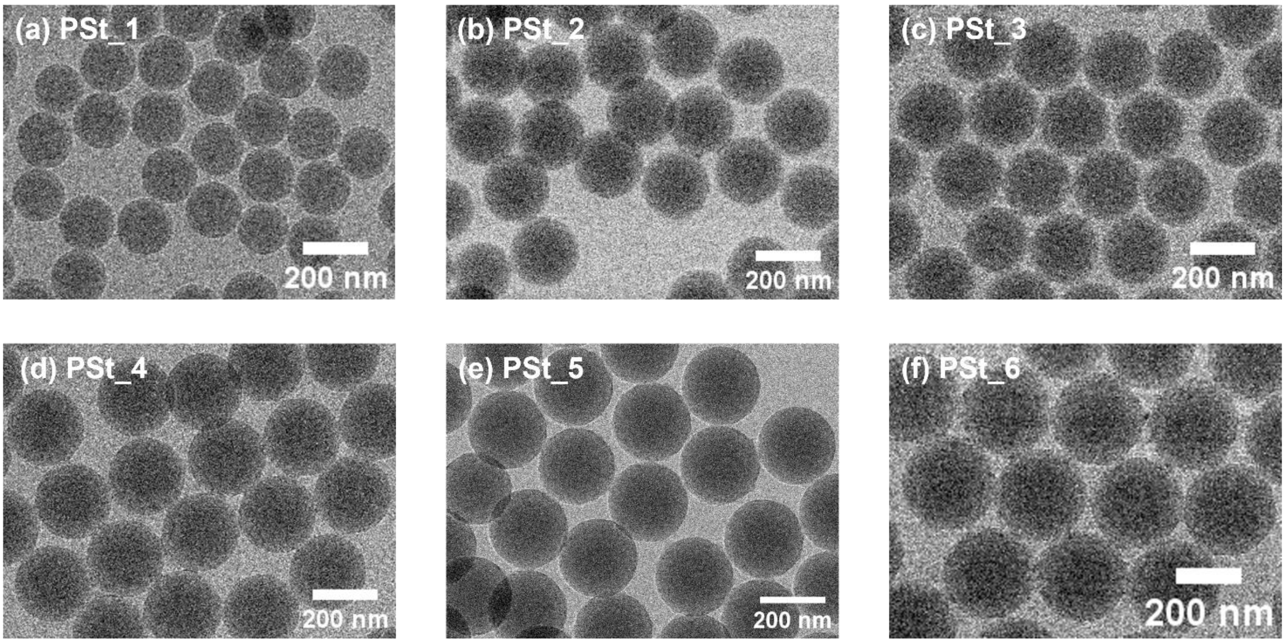

Figure S2. TEM images of PSt particles from Table S1.

Synthesis of PSt@PDA particles

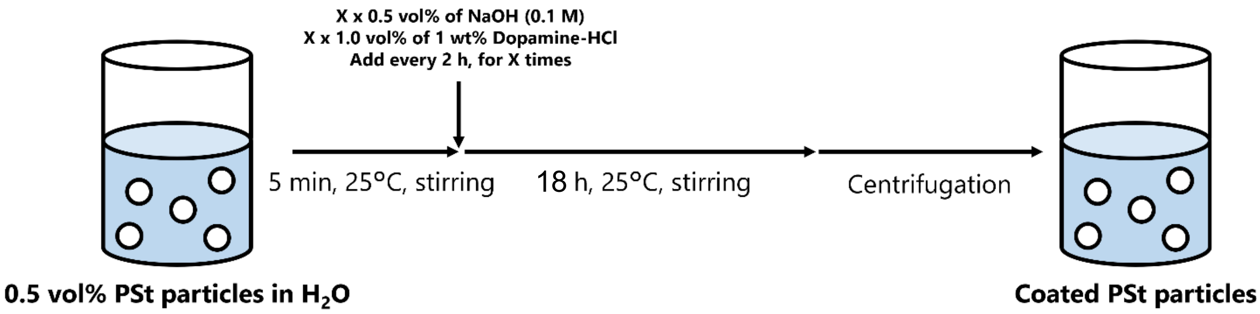

Figure S3. Procedure for coating PSt particles to make PSt@PDA particles. For PSt_4@PDA_1 (used in PSt@PDA-1/Silica SPs in Figure 6) and PSt_4@PDA_2 (used in PSt@PDA-2/Silica SPs in Figure 6), dopamine-HCl and NaOH were added X=1 and X=3 times, respectively. This corresponds to 0.132 and 0.396 mg DA-HCl/mg PSt particles. However, we note that the amount of mg DA-HCl/mg PSt particles is not comparable to mg DA-HCl/mg SPs due to the different synthesis conditions.

Procedure to produce silica-supported supraparticles

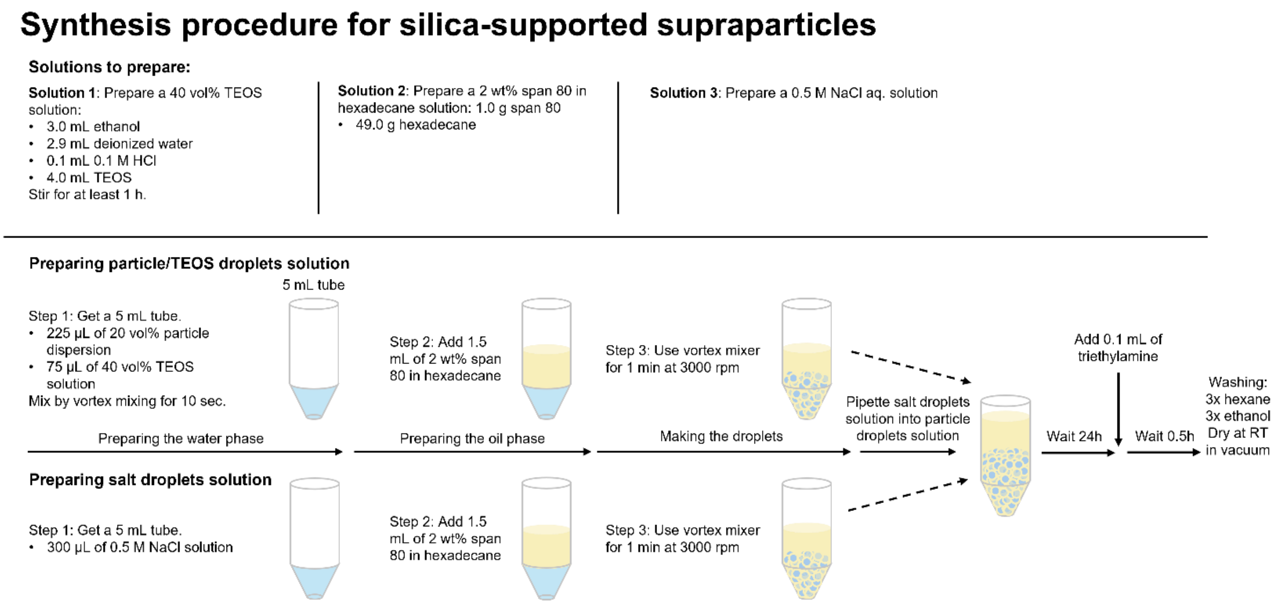

Figure S4. Procedure for silica-supported supraparticle production.

Table S2. Synthesis conditions for each supraparticle sample and Figures in which they appear in the main manuscript. The oil phase was always 1.5 mL hexadecane (2 wt% Span 80) except for Figure 2 in which 0.25 mL hexadecane (2 wt% Span 80) was added.

Fig.	Label in Fig.	SP label	Water phase
2	3:1 ratio		0.0375 mL 10 vol% PSt_1 + 0.0125 mL 20 vol% TEOS solution
2	1:1 (2.0 M)		0.025 mL 10 vol% PSt_1 + 0.025 mL 20 vol% TEOS solution
2	2:1 (2.0 M)		0.0333 mL 10 vol% PSt_1 + 0.0167 mL 20 vol% TEOS solution
2	3:1 (2.0 M)		0.0375 mL 10 vol% PSt_1 + 0.0125 mL 20 vol% TEOS solution
2	2:1 (0.5 M)		0.0333 mL 10 vol% PSt_1 + 0.0167 mL 20 vol% TEOS solution
3	PSt/Silica SP	12-4SP4	0.225 mL 20 vol% PSt_3 + 0.075 mL 40 vol% TEOS solution

4	5:1 ratio	11-27SP1	0.3 mL 20 vol% PSt_1 + 0.06 mL 40 vol% TEOS solution
4	2.5:1 ratio	11-27SP2	0.25 mL 20 vol% PSt_1 + 0.1 mL 40 vol% TEOS solution
4	1:1 ratio	11-27SP4	0.175 mL 20 vol% PSt_1 + 0.175 mL 40 vol% TEOS solution
5	(PSt/Silica) SPs	12-4SP1	0.225 mL 20 vol% PSt_5 + 0.075 mL 40 vol% TEOS solution
6	(PSt/Silica) SP and PDA-coated (PSt/Silica) SPs	12-4SP5	0.225 mL 20 vol% PSt_4 + 0.075 mL 40 vol% TEOS solution
6	PSt@PDA-1/Silica	12-4SP12	0.225 mL 20 vol% PSt_4@PDA_1 + 0.075 mL 40 vol% TEOS solution
6	PSt@PDA-2/Silica	12-4SP13	0.225 mL 20 vol% PSt_4@PDA_2 + 0.075 mL 40 vol% TEOS solution
7	Blue SPs	11-27SP2	0.25 mL 20 vol% PSt_1 + 0.1 mL 40 vol% TEOS solution
7	Teal SPs	12-1SP2	0.25 mL 20 vol% PSt_2 + 0.1 mL 40 vol% TEOS solution
7	Green SPs	12-4SP4	0.225 mL 20 vol% PSt_3 + 0.075 mL 40 vol% TEOS solution
7	Yellow SPs	12-4SP5	0.225 mL 20 vol% PSt_4 + 0.075 mL 40 vol% TEOS solution
7	Orange SPs	12-4SP1	0.225 mL 20 vol% PSt_5 + 0.075 mL 40 vol% TEOS solution
7	Red SPs	12-4SP6	0.225 mL 20 vol% PSt_6 + 0.075 mL 40 vol% TEOS solution
8	PSt@PDA/Silica	12-4SP12	0.225 mL 20 vol% PSt_4@PDA_1 + 0.075 mL 40 vol% TEOS solution
8	(PSt/Silica)@PDA*[1]	12-1SP5-0.5*	0.25 mL 20 vol% PSt_1 + 0.1 mL 40 vol% TEOS solution
8	(PSt/Silica)@PDA@PEI	12-4SP4	0.225 mL 20 vol% PSt_3 + 0.075 mL 40 vol% TEOS solution

S12	12-4SP9	12-4SP9	0.225 mL 20 vol% PSt_1@PDA_3*2 + 0.075 mL 40 vol% TEOS solution
S12	12-4SP10	12-4SP10	0.225 mL 20 vol% PSt_1@PDA_1 + 0.075 mL 40 vol% TEOS solution
S12	12-4SP11	12-4SP11	0.225 mL 20 vol% PSt_3@PDA_1 + 0.075 mL 40 vol% TEOS solution
S12	12-4SP12	12-4SP12	0.225 mL 20 vol% PSt_4@PDA_1 + 0.075 mL 40 vol% TEOS solution
S12	12-4SP13	12-4SP13	0.225 mL 20 vol% PSt_4@PDA_2 + 0.075 mL 40 vol% TEOS solution
S13	(PSt/Silica)@PDA-0.5 SPs	12-1SP4	0.25 mL 20 vol% PSt_1 + 0.1 mL 40 vol% TEOS solution
S13	PSt@PDA-1/Silica SPs	12-4SP9	0.225 mL 20 vol% PSt_1@PDA_0.5 + 0.075 mL 40 vol% TEOS solution
S13	PSt@PDA-2/Silica SPs	12-4SP10	0.225 mL 20 vol% PSt_1@PDA_1 + 0.075 mL 40 vol% TEOS solution

*1Coated with 0.5 mg DA-HCL per mg of SPs.

*2This corresponds to 0.066 mg DA-HCl/mg PSt particles.

Shrinking droplets with 2.0 M NaCl aq. droplets

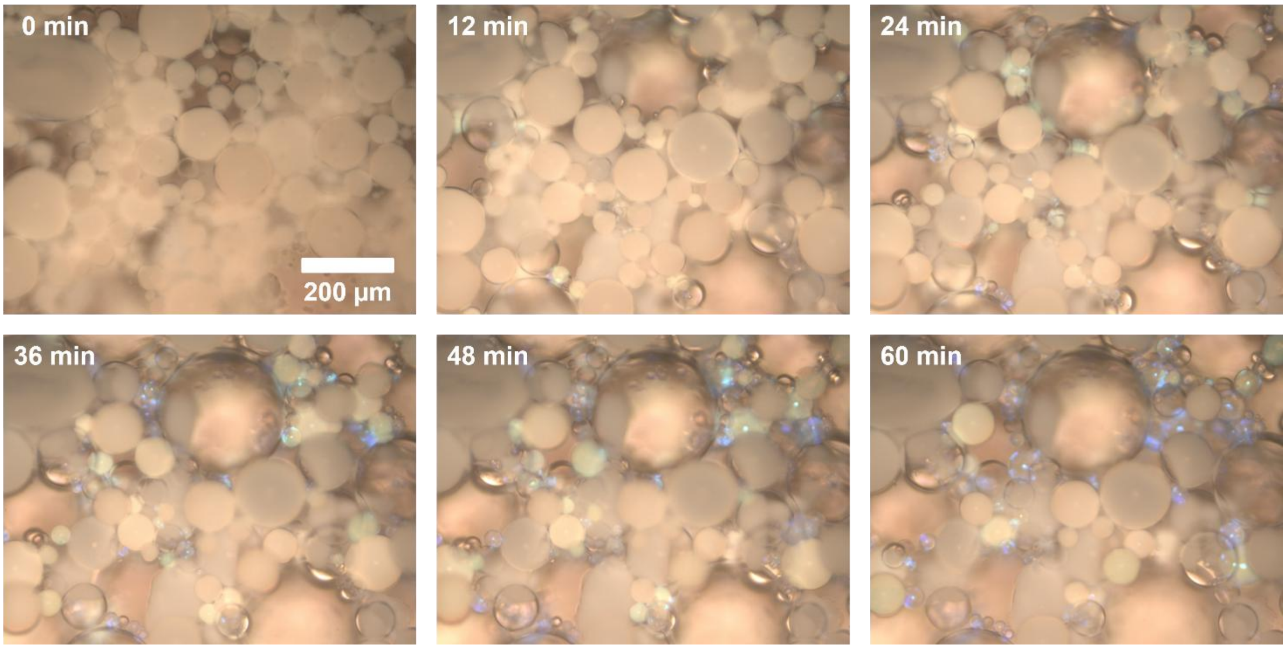

Figure S5. Optical microscope snapshots taken during the osmosis-induced shrinking process with particle droplets containing a 3:1 ratio between 10 vol% PSt particle dispersion and 20 vol% TEOS solution. The particle droplets were mixed with 2.0 M NaCl aq. droplets in a 1:1 volume ratio.

Images of supraparticles fabricated using 2.0 M NaCl aq. droplets.

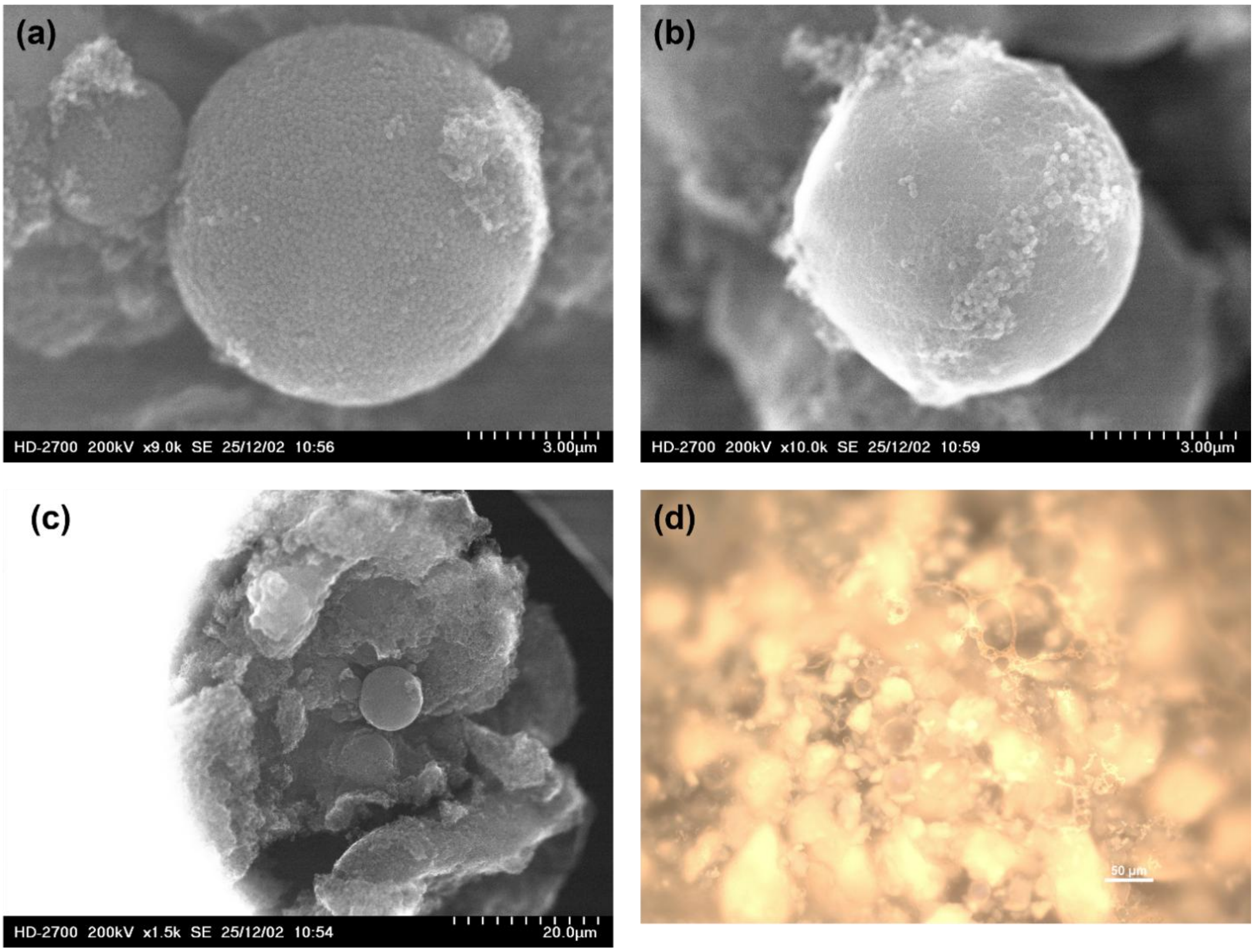

Figure S6. (a-c) TEM and (d) optical microscope images of silica-supported SPs using the same conditions as in Figure 4(b,e) except they were osmotically shrunk using 2.0 M NaCl droplets. The shrinking was so fast that most of the emulsion became unstable and not many supraparticles formed.

Physisorption & thermogravimetric analysis of PSt@PDA/Silica SPs

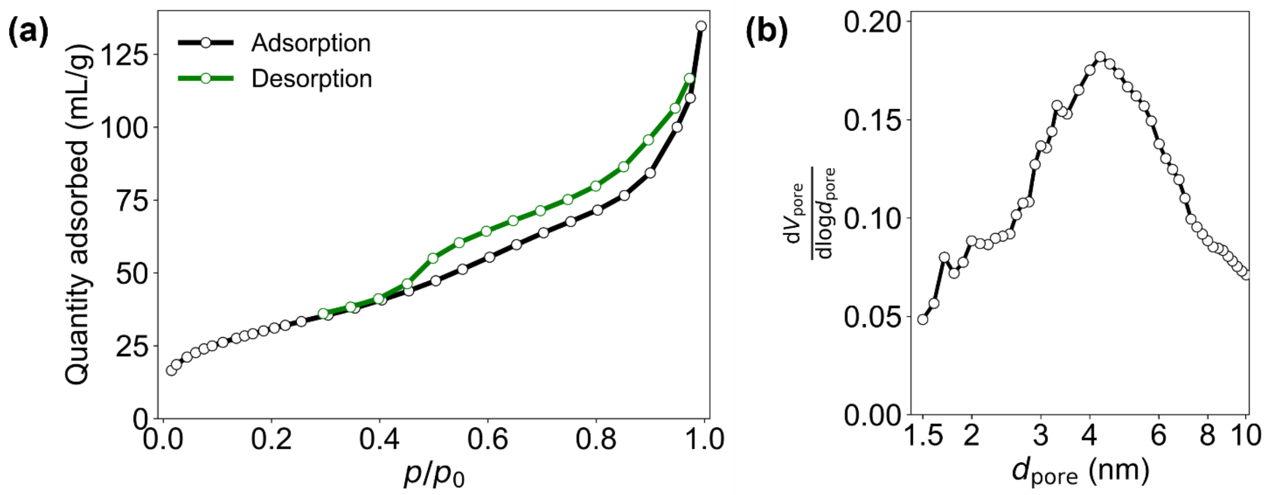

Figure S7. Nitrogen physisorption results for PSt@PDA/Silica SPs. (a) Nitrogen adsorption/desorption curves. (b) Pore size distribution. The BET surface area was 110 m^2/g.

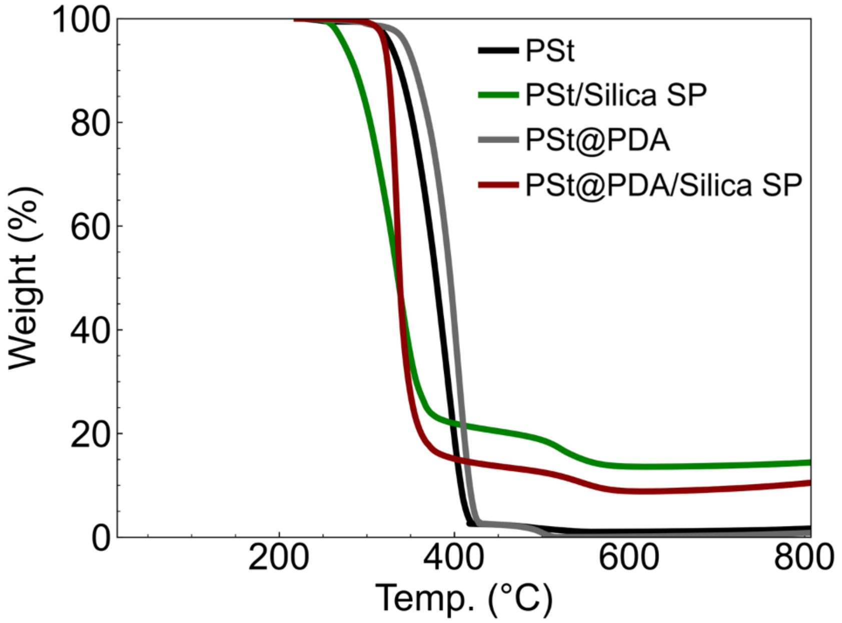

Figure S8. TG results for PSt@PDA and PSt@PDA/Silica SPs compared to PSt/Silica SPs.

Structural and Optical Characterization

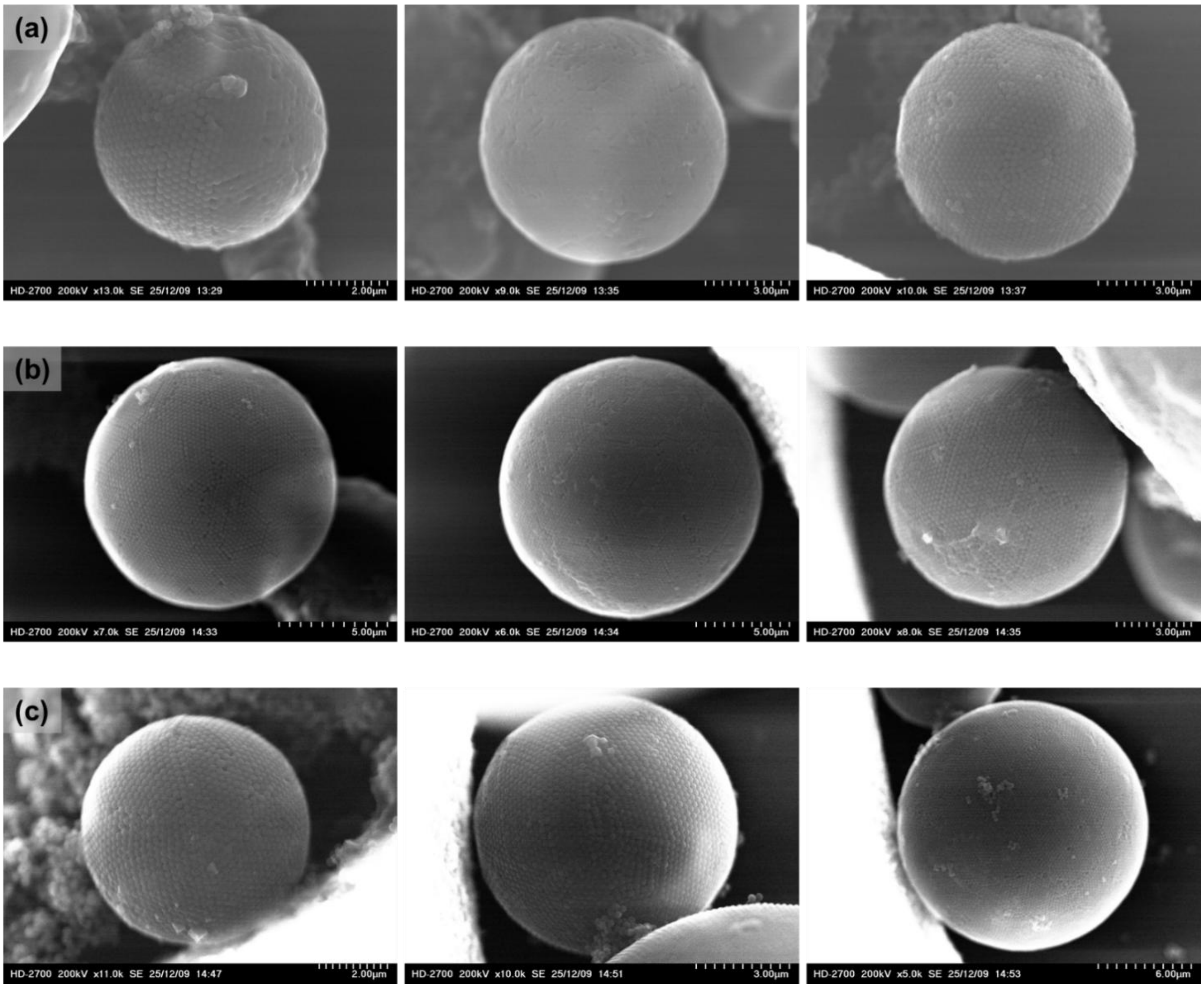

Figure S9. TEM images of (a) PSt/Silica SPs from Figure 6a, (b) PSt@PDA-1/Silica SPs from Figure 6c, and (c) PSt@PDA-2/Silica SPs from Figure 6d. Hexagonal arrangements of PSt particles could be seen at the surface of SPs for (a) and (b). As for (c), a wide layer of aligned PSt particles around the SPs could be seen.

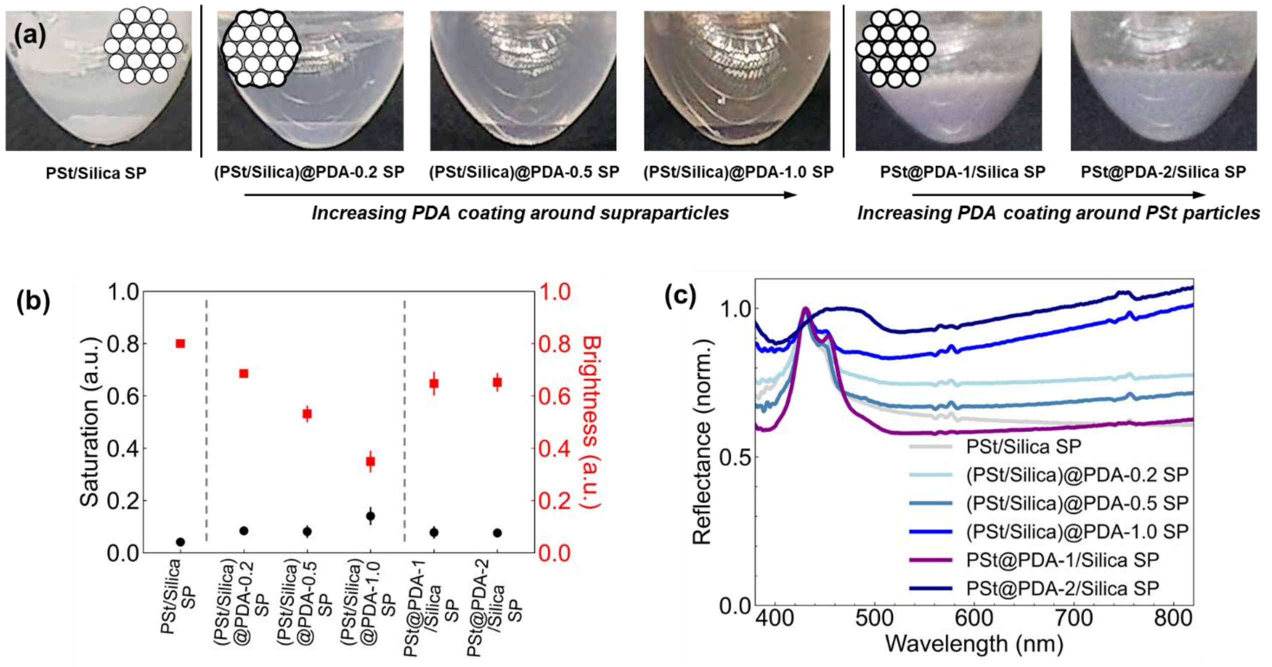

Figure S10. Optical properties of supraparticles coated with polydopamine post-assembly or supraparticles formed using polydopamine-coated PSt building blocks for smaller building blocks than Fig. 6 in the main text. (a) Photos of SPs with varying amounts of polydopamine coated around supraparticles after formation or coated around PSt particles before supraparticle formation. (b) Saturation and brightness values derived from the photos in (a). (c) Reflectance spectra for particles with varying amounts and placement of polydopamine.

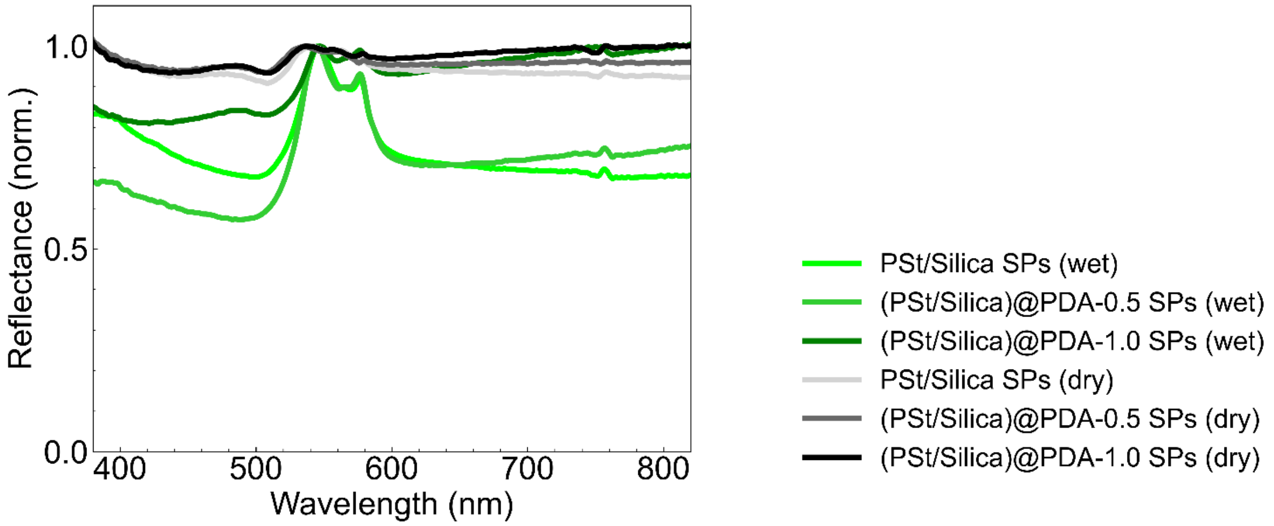

Figure S11. Reflectance spectra of SPs in the wet (in water) and dry state.

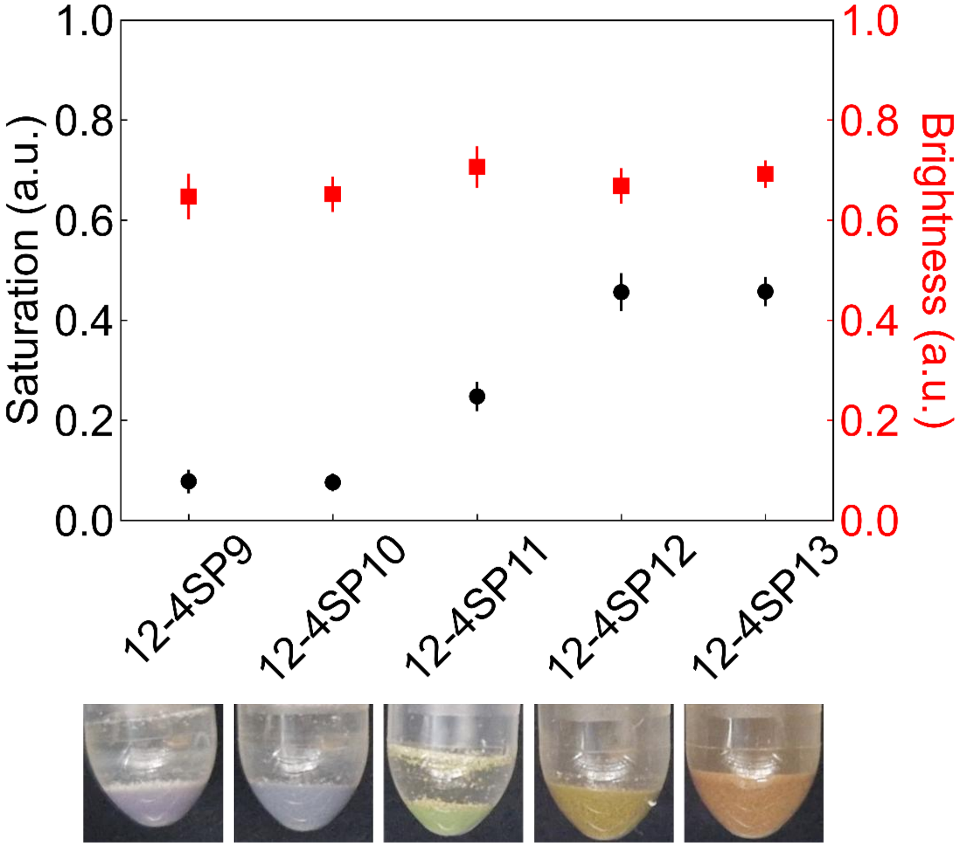

Figure S12. Saturation and brightness for PSt@PDA/Silica SPs of different colors.

SAXS of (PSt/Silica)@PDa & PSt@PDA/Silica SPs

For these measurements, PSt@PDA/Silica and (PSt/Silica)@PDA SPs with PSt-1 building blocks (See Table S1) were used. PSt@PDA-1/Silica and PSt@PDA-2/Silica SPs differ in the thickness of the PDA coating around the PSt-1 building blocks. PSt@PDA-2/Silica SPs showed a significantly broadened reflection peak, so we wanted to confirm if this was due to the structure by using SAXS. The assembled structures of supraparticles were confirmed using a benchtop-type small angle X-ray scattering system (NANOPIX mini, RIGAKU, Japan). Glass capillaries (Mark tubes made of glass no. 50, Hilgenberg, Germany) were used to measure samples dispersed in water. After introducing samples, capillaries were sealed with UV resin in order not to evaporate water during measurements. To subtract the scattering from backgrounds, the 1D profile of a capillary filled with water was measured in advance. The profiles were smoothed using a Savitzky–Golay filter (window size: 100 points, polynomial order: 2).

The scattering intensity $I(q)$ is given by the product of the form factor $P(q)$ and the structure factor $S(q)$. For spherical particles, $P(q)$ is given as follows.[1,2]

$$P(q) = \frac{9[\sin(qr) - qr\cos(qr)]^2}{(qr)^6}, \qquad \text{Eq. S1}$$

where q is the scattering vector [nm^{-1}] and r is the radius of particles [nm]. If the volume fraction of particles or the regularity of the arrangement of particles is low, $I(q) \sim P(q)$. On the other hand, if particles form an ordered structure, $I(q)$ shows some peaks owing to $S(q)$. the scattering peaks derived from $\{hkl\}$ planes appear at $q_{hkl} = 2\pi/d_{hkl}$. where $d_{hkl} = 2\sqrt{2}r/\sqrt{h^2+k^2+l^2}$ for face-centered cubic (fcc) crystal structure.[3] Figure shows SAXS profiles of silica-supported supraparticles and the form factor $P(q)$ calculated assuming r = 85 nm. The dotted lines indicate scattering vectors from fcc$\{hkl\}$ planes (also assuming r = 85 nm).

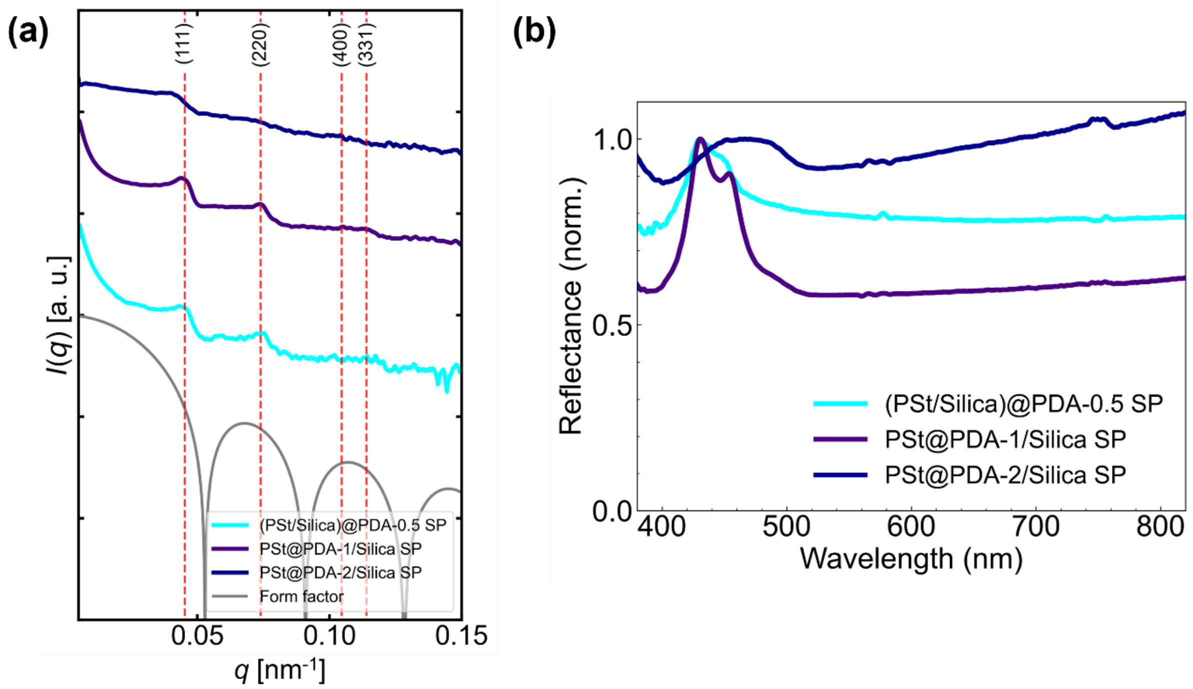

Figure S13. (a) SAXS measurement of various SPs. (b) Reflectance measurement of the same SPs as (a).

PSt@PDA-2/Silica SP showed a peak at $q \sim 0.3$ nm^{-1} unlike $P(q)$; however, it was broad and did not correspond to q_{111}. In addition, PSt@PDA-2/Silica SP did not show any peaks in larger q. Therefore, PSt@PDA-2/Silica SP likely formed randomly packed structures. On the other hand, (PSt/Silica)@PDA-0.5 SP and PSt@PDA-1/Silica SP showed some peaks, which were well matched with q_{111}, q_{220}, q_{400}, and q_{331}, respectively. Based on systematic absences, {111}, {200}, {220}, {311}, {222}, {400},

{331}, {420}... are able to be observed in X-ray scattering measurements.[4,5] Some scattering peaks like fcc{200}, {311}, and {220} could not be observed in Figure S13 likely because they were also overlapped with the valley of $P(q)$ in our case. It should also be noted that some peaks could also be attributed to the hexagonal close-packed (hcp) lattice structure; for example, scattering from hcp(002) and hcp(110) are completely overlapped with fcc(111) and fcc(220), respectively. Therefore, it is difficult to determine the lattice structure of the samples quantitatively. Nevertheless, we can conclude that (PSt/Silica)@PDA-0.5 SP and PSt@PDA-1/Silica SP at least formed random hexagonal close-packed (rhcp) crystal structures, whereas PSt@PDA-2/Silica SP formed less ordered structures. The SAXS measurements confirmed that the reflection peak is a good measure for the structural order of the SPs.

Zeta potential of PSt & PSt@PDA building blocks

The zeta potential of the PSt and PSt@PDA building blocks were measured using a Malvern Zetasizer Ultra.

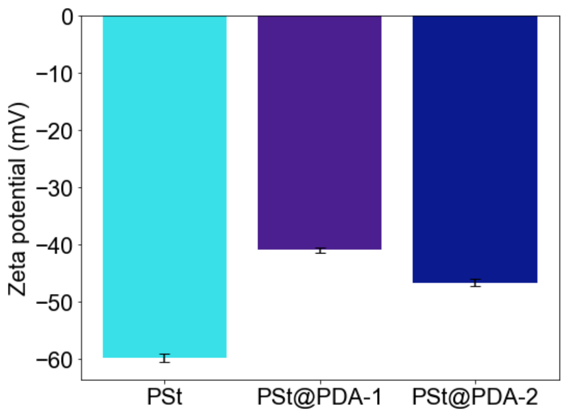

Figure S14. Zeta potential of the various particles used as building blocks for SPs in this work.

Evaluation of stability of silica-supported SPs in different pH

To highlight the stability of the SPs, 20 μL of concentrated SP suspension was pipetted into 5-mL tubes containing 2 mL of various pH aqueous solutions and shaken at 1000 rpm for 24 h. Subsequently, the tubes were centrifuged at 14800 rpm for 10 s and observed under the optical microscope and scanning electron microscope (Figure S15). Most of silica-supported SPs retained their spherical structures and optical reflections. However, SEM images, as displayed in Figure S16, show that at pH 13.2 a lot of silica was deposited on the supraparticles after drying the SEM sample. This indicates that the silica starts to dissolve at such pH, which is expected. The SPs are therefore unlikely to stay stable at this pH forever.

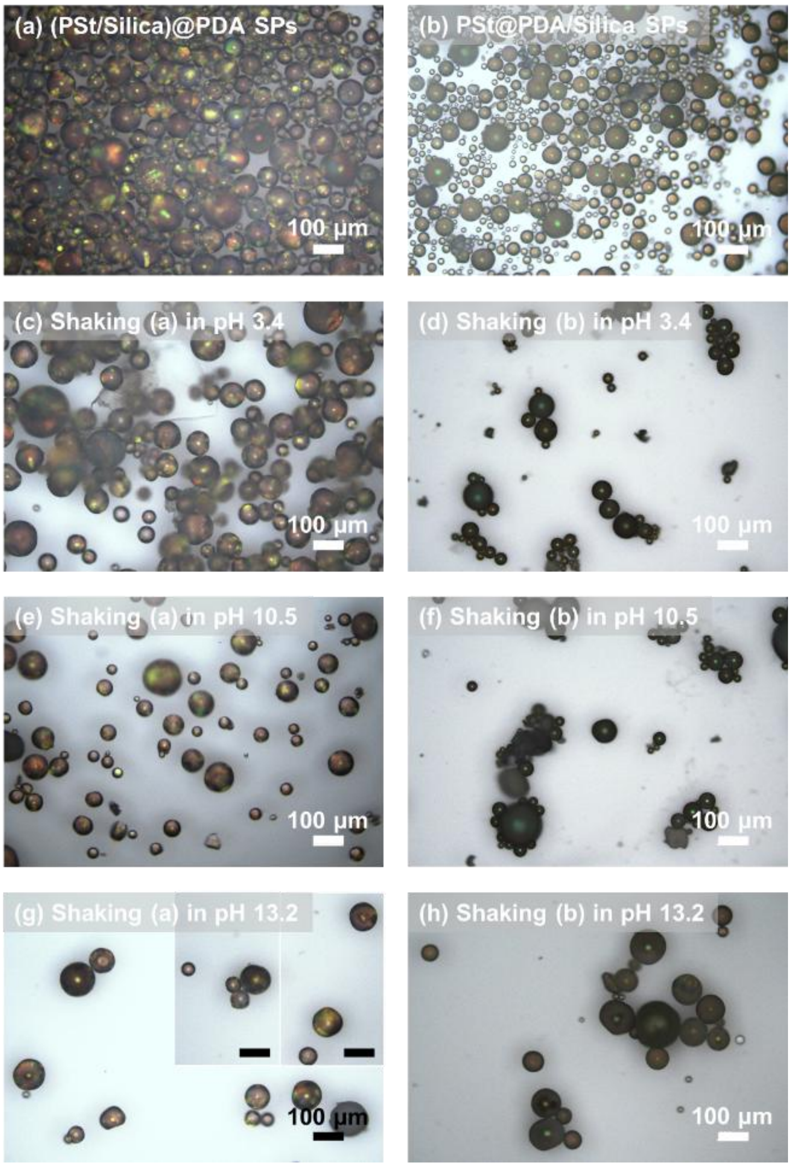

Figure S15. Optical images of (PSt/Silica)@PDA SPs (a,c,e,g) and PSt@PDA/Silica SPs (b,d,f,h) before (a,b) and after shaking at various pH conditions (c-h).

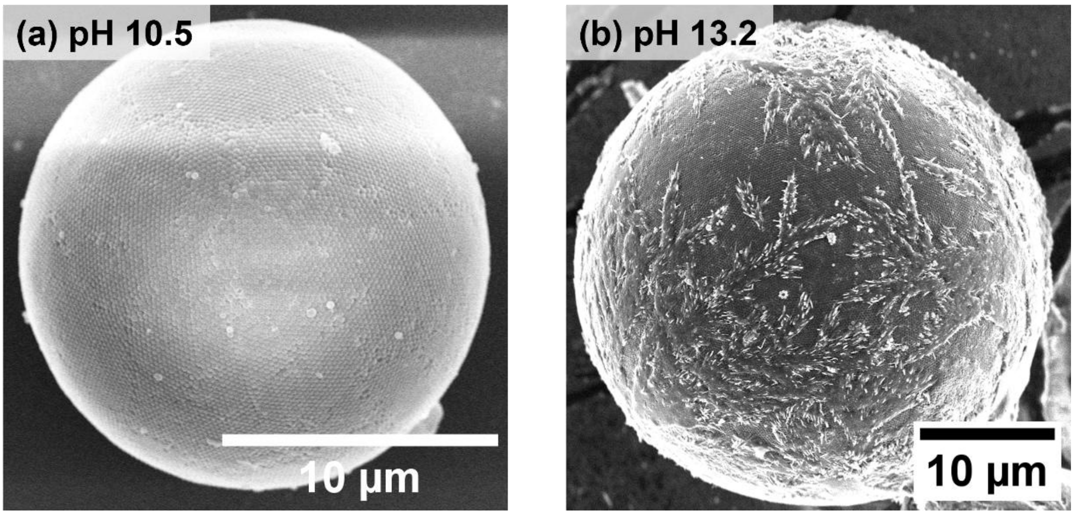

Figure S16. SEM images of PSt@PDA/Silica SPs after shaking for 24 hours at pH 10.5 and pH 13.2.

Supporting Movie Captions

1. Time lapse of osmotic shrinking of particle/TEOS droplets (3:1 ratio of 10 vol% particle dispersion to 20 vol% TEOS solution) mixed with 2.0 M NaCl aq. droplets in a 1:1 ratio.
2. Time lapse of osmotic shrinking of particle/TEOS droplets (2:1 ratio of 10 vol% particle dispersion to 20 vol% TEOS solution) mixed with 2.0 M NaCl aq. droplets in a 1:1 ratio.
3. Time lapse of osmotic shrinking of particle/TEOS droplets (1:1 ratio of 10 vol% particle dispersion to 20 vol% TEOS solution) mixed with 2.0 M NaCl aq. droplets in a 1:1 ratio.
4. Time lapse of osmotic shrinking of particle/TEOS droplets (3:1 ratio of 10 vol% particle dispersion to 20 vol% TEOS solution) mixed with 0.5 M NaCl aq. droplets in a 1:1 ratio.
5. Optical microscopy movie of (PSt/Silica)@PDA@PEI SPs in deionized water under a 5.2 V/mm electric field which points towards the left.
6. Optical microscopy movie of (PSt/Silica)@PDA SPs in deionized water under a 5.2 V/mm electric field which points towards the left.
7. Optical microscopy movie of PSt@PDA/Silica SPs in deionized water under a 5.2 V/mm electric field which points towards the right.
8. Macroscopic movie of (PSt/Silica)@PDA@PEI SPs in deionized water under a 5.2 V/mm electric field which switches direction multiple times.

References

(1) Guinier, A.; Fournet, G. Small-Angle Scattering of X-Rays; Wiley, 1955.

(2) Pedersen, J. S. Analysis of Small-Angle Scattering Data from Colloids and Polymer Solutions: Modeling and Least-Squares Fitting; 1997; Vol. 70, pp 171–210. https://doi.org/10.1016/S0001-8686(97)00312-6.

(3) Montanarella, F.; Geuchies, J. J.; Dasgupta, T.; Prins, P. T.; Van Overbeek, C.; Dattani, R.; Baesjou, P.; Dijkstra, M.; Petukhov, A. V.; Van Blaaderen, A.; Vanmaekelbergh, D. Crystallization of Nanocrystals in Spherical Confinement Probed by in Situ X-Ray Scattering. Nano Lett. 2018, 18 (6), 3675–3681. https://doi.org/10.1021/acs.nanolett.8b00809.

(4) Farrell, D. F.; Ijiri, Y.; Kelly, C. V.; Borchers, J. A.; Rhyne, J. J.; Ding, Y.; Majetich, S. A. Small Angle Neutron Scattering Study of Disordered and Crystalline Iron Nanoparticle Assemblies. J. Magn. Magn. Mater. 2006, 303 (2), 318–322. https://doi.org/10.1016/J.JMMM.2006.01.219.

(5) Wu, L.; Willis, J. J.; McKay, I. S.; Diroll, B. T.; Qin, J.; Cargnello, M.; Tassone, C. J. High-Temperature Crystallization of Nanocrystals into Three-Dimensional Superlattices. Nature 2017, 548 (7666), 197–201. https://doi.org/10.1038/nature23308.
